# Supplementary material for: Childhood tuberculosis outcomes and factors associated with unsuccessful treatment outcomes in selected public hospitals of Lusaka Zambia from 2015 to 2019
Source: PLOS Glob Public Health. 2024 Oct 11;4(10):e0002591. doi: 10.1371/journal.pgph.0002591 (PMC11469523; doi:10.1371/journal.pgph.0002591)
Supplement: S1 Table — (DOCX) [file pgph.0002591.s001.docx]

**S1 Table:** Evaluation status of treatment outcome for children treated for TB by demographic and clinical characteristics (2015 - 2019)

| Characteristics | Treatment outcome evaluation status  n (%) | | Total cases | Chi-square  P-value |
| --- | --- | --- | --- | --- |
|  | **Outcome not Evaluated** | **Outcome evaluated** |  |  |
| Overall | 1036 (40.9) | 1495 (59.1) | 2,531 |  |
| Age |  |  |  |  |
| Less than 5 years | 591 (44.7) | 732 (55.3) | 1,323 | <0.001* |
| 5 – 14 years | 445 (36.9) | 760 (63.1) | 1,205 |  |
| Sex |  |  |  |  |
| Female | 465 (38.9) | 731 (61.1) | 1,196 | 0.061 |
| Male | 565 (42.5) | 763 (57.4) | 1,328 |  |
| Infection site |  |  |  |  |
| Extra Pulmonary | 246 (39.0) | 385 (61.0) | 631 | 0.262 |
| Pulmonary | 786 (41.5) | 1107 (58.5) | 1,893 |  |
| Patient Type |  |  |  |  |
| New Patient | 1010 (41.2) | 1442 (58.8) | 2,452 | 0.106 |
| Retreatment | 21 (31.3) | 46 (68.7) | 67 |  |
| HIV status |  |  |  |  |
| Negative | 532 (37.7) | 878 (62.3) | 1,410 | <0.001* |
| Positive | 445 (42.9) | 592 (57.1) | 1,037 |  |
| Unknown | 59 (70.2) | 25 (29.8) | 84 |  |
| Year |  |  |  |  |
| 2015 | 49 (21.1) | 183 (78.9) | 232 | <0.001 |
| 2016 | 99 (36.8) | 170 (63.2) | 269 |  |
| 2017 | 301 (47.8) | 328 (52.1) | 629 |  |
| 2018 | 395 (48.5) | 420 (51.5) | 815 |  |
| 2019 | 192 (32.8) | 394 (67.2) | 586 |  |
| Facility |  |  |  |  |
| Chawama | 20 (6.6) | 282 (93.4) | 302 | <0.001 |
| Chelstone | 19 (20.6) | 73 (79.3) | 92 |  |
| Chilenje | 12 (10.9) | 98 (89.1) | 110 |  |
| Chipata | 12 (10.6) | 101 (89.4) | 113 |  |
| Kanyama | 22 (6.8) | 299 (93.1) | 321 |  |
| Matero | 211 (38.2) | 342 (61.8) | 553 |  |
| LMUTH | 94 (75.2) | 31 (24.8) | 125 |  |
| UTH | 646 (70.6) | 269 (29.4) | 915 |  |
| ** Statistically significant at 5% significance level, CI Confidence Interval, TB Tuberculosis, n Frequency, % Percentage, HIV Human Immune Virus* | | | | |
