# Supplementary material for: Childhood tuberculosis outcomes and factors associated with unsuccessful treatment outcomes in selected public hospitals of Lusaka Zambia from 2015 to 2019
Source: PLOS Glob Public Health. 2024 Oct 11;4(10):e0002591. doi: 10.1371/journal.pgph.0002591 (PMC11469523; doi:10.1371/journal.pgph.0002591)
Supplement: Attachment — Submitted filename: PGPH-D-23-01445_R2_reviewer_KS.pdf [file pgph.0002591.s004.pdf]

PLOS Global Public Health

Childhood Tuberculosis Outcomes and Factors Associated with Unsuccessful Treatment Outcomes in Selected Public Hospitals of Lusaka Zambia from 2015 to 2019

--Manuscript Draft--

**Manuscript Number:**	PGPH-D-23-01445R2
**Article Type:**	Research Article
**Full Title:**	Childhood Tuberculosis Outcomes and Factors Associated with Unsuccessful Treatment Outcomes in Selected Public Hospitals of Lusaka Zambia from 2015 to 2019
**Short Title:**	Factors Associated with Unsuccessful Treatment Outcomes
**Corresponding Author:**	Dennis Ngosa, MSc. Epidemiology<br>University of Zambia<br>Lusaka, Lusaka ZAMBIA
**Order of Authors:**	Dennis Ngosa, MSc. Epidemiology
	Joseph Lupenga, MSc. Epidemiology
**Keywords:**	Keywords: Treatment outcomes, tuberculosis, Unsuccessful treatment outcomes
**Abstract:**	Treatment outcomes of tuberculosis in children are rarely evaluated. Childhood tuberculosis has been a low priority for tuberculosis programs due to difficulties in diagnosis and treatment. This study evaluated childhood tuberculosis outcomes and factors associated with unsuccessful treatment outcomes in selected public Hospitals in Lusaka, Zambia from 2015 to 2019.<br>This was a retrospective cross-sectional study conducted in 8 public Hospitals in Lusaka. All children aged 0 – 14 years, treated with tuberculosis were included. The WHO tuberculosis treatment outcomes were grouped into successful treatment outcome (cured, treatment completed) and unsuccessful treatment outcome (death, loss to follow-up, failure). Taking unsuccessful treatment outcome as the outcome variable, unadjusted and adjusted logistic regressions were performed. All analyses were done at a 95% confidence interval.<br>Out of 2,531 participants, majority were 0 to 4 years old (52.3%), males (52.6%), HIV-negative (55.7%), and had pulmonary tuberculosis (75%). About 80% of children, were treated for TB based on clinical diagnosis. Bacteriological tests were performed on 20% of children, where 55% had positive bacteriological results. Bacteriologically confirmed TB was higher in children over 5 years (68.4%), pulmonary TB (60.1%), and retreatment (83.3%). Overall, 6.3% of patients were cured, 49.7% completed treatment, 0.9% lost to follow-up, 1.8% died, 0.3% failed treatment and 40.9% were not evaluated. The overall unsuccessful treatment outcome was 5.1% with a successful treatment outcome of 94.1% after the not-evaluated children were dropped. Extrapulmonary tuberculosis was associated with unsuccessful treatment outcomes (AOR 1.64; 95 % CI: 1.02 – 2.62).<br>A high proportion of children were not evaluated for treatment outcomes. The successful treatment outcome met the World Health Organization's threshold goal of 90% after children who were not evaluated were dropped. Children with extrapulmonary tuberculosis should be targeted as a high-risk group to improve treatment outcomes.
**Additional Information:**	
**Question**	**Response**
**Financial Disclosure**<br><br>Enter a financial disclosure statement that describes the sources of funding for the	This was not funded research

work included in this submission and the role the funder(s) played. This includes grants and any commercial funding of the work or authors.<br><br>This statement will be typeset if the manuscript is accepted for publication.<br><br>*Please review the submission guidelines and the instructions link below for detailed requirements and guidance.*	
**Competing Interests**<br><br>On behalf of all authors, disclose any competing interests that could be perceived to bias this work.<br><br>This statement will be typeset if the manuscript is accepted for publication.<br><br>*Please review the instructions link below and PLOS Global Public Health's competing interests policy to determine what information must be disclosed at submission.*	The authors declare that they have no competing interests.
**Data Availability**<br><br>Before publication, Authors are required to make fully available and without restriction all data underlying their findings. Please see our PLOS Data Policy page for detailed information on this policy.<br><br>A **Data Availability Statement**, detailing where the data can be accessed, is required at first submission. Insert your Data Availability Statement in the box below.<br><br>Please see the data reporting section of our submission guidelines for instructions	The dataset that was analyzed for this study can be accessed using the following link: https://figshare.com/s/9fee2b9fb8f0aed4b01c

on what you need to include in your Data Availability Statement.<br><br>This statement will be typeset if the manuscript is accepted for publication.<br><br>*PLOS allows rare exemptions to address legal and ethical concerns. If you have legal or ethical restrictions, please use the box below to detail these in full sentences for the Journal team to consider.*	

Manuscript Click here to access/download;Manuscript;Manuscript.docx

Childhood Tuberculosis Outcomes and Factors Associated with Unsuccessful Treatment Outcomes in Selected Public Hospitals of Lusaka Zambia from 2015 to 2019

Dennis Ngosa [1,2*¶], Joseph Lupenga[1¶]

1 The University of Zambia, Department of Epidemiology and Biostatistics, School of Public Health, Lusaka, Zambia

2 Analysis Department, Centre for Infectious Diseases Research in Zambia, Lusaka, Zambia

***Corresponding Author:** *dennisngosam@gmail.com*

¶ These authors contributed equally to this work

Abstract

Treatment outcomes of tuberculosis in children are rarely evaluated. Childhood tuberculosis has been a low priority for tuberculosis programs due to difficulties in diagnosis and treatment. This study evaluated childhood tuberculosis outcomes and factors associated with unsuccessful treatment outcomes in selected public Hospitals in Lusaka, Zambia from 2015 to 2019.

This was a retrospective cross-sectional study conducted in 8 public Hospitals in Lusaka. All children aged 0 – 14 years, treated with tuberculosis were included. The WHO tuberculosis treatment outcomes were grouped into successful treatment outcome (cured, treatment completed) and unsuccessful treatment outcome (death, loss to follow-up, failure). Taking unsuccessful treatment outcome as the outcome variable, unadjusted and adjusted logistic regressions were performed. All analyses were done at a 95% confidence interval.

Out of 2,531 participants, majority were 0 to 4 years old (52.3%), males (52.6%), HIV-negative (55.7%), and had pulmonary tuberculosis (75%). About 80% of children, were treated for TB based on clinical diagnosis. Bacteriological tests were performed on 20% of children, where 55% had positive bacteriological results. Bacteriologically confirmed TB was higher in children over 5 years (68.4%), pulmonary TB (60.1%), and retreatment (83.3%). Overall, 6.3% of patients were cured, 49.7% completed treatment, 0.9% lost to follow-up, 1.8% died, 0.3% failed treatment and 40.9% were not evaluated. The overall unsuccessful treatment outcome was 5.1% with a successful treatment outcome of 94.1% after the not-evaluated children were dropped. Extrapulmonary tuberculosis was associated with unsuccessful treatment outcomes (AOR 1.64; 95 % CI: 1.02 – 2.62).

A high proportion of children were not evaluated for treatment outcomes. The successful treatment outcome met the World Health Organization's threshold goal of 90% after children who were not evaluated were dropped. Children with extrapulmonary tuberculosis should be targeted as a high-risk group to improve treatment outcomes.

Keywords: *Treatment outcomes, tuberculosis, Unsuccessful treatment outcomes*

Introduction

Before the coronavirus (COVID-19) pandemic, tuberculosis (TB) was the most common infectious cause of death, surpassing HIV/AIDS (1). An estimated 10.8 million new cases of the illness and over 1.6 million estimated TB deaths were recorded globally in 2021 (1). Out of these, 6 million were men, 3.4 million were women and 1.2 million were children (1).

Although TB affects adults more frequently than children, children are often seen as a specific risk group. This is because TB in children can be challenging to diagnose and treat (1–3). Furthermore, TB in children and adolescents is frequently overlooked by health providers and has been a low priority for national TB programs (4,5).

Ending the TB epidemic by 2030 is one of the Sustainable Development Goals' health targets (6) (6). Zambia is among the countries that have committed to the end TB by 2030 strategy, which includes implementing the 90-90-90 TB strategy (8). One of the three main goals is to achieve a 90 percent treatment success rate among those who have been identified as needing treatment (7). However, the TB treatment outcome data (successful or unsuccessful treatment outcomes) for children 0–14 years are not reported in most countries (8). A systematic review on treating children

with TB reported that children have been left behind and neglected as data on many aspects of the treatment of childhood TB is still scarce, and the paucity of high-quality data is concerning (9).

Previous studies have shown that the outcome of TB treatment in children varies with age, sex, HIV status, and type of TB (10,11). Several factors put children who are diagnosed with TB disease and initiated on treatment at higher risk of experiencing unsuccessful treatment outcomes, including treatment failure, death, or being lost to follow-up. Sex, age, and type of TB have been reported to be associated with unsuccessful treatment outcomes (10,12). However, other studies argued that sex, age, and type of TB were not associated with unsuccessful treatment outcomes. (13).

Monitoring treatment outcomes in children with TB and understanding the factors associated with unsuccessful treatment outcomes is critical in achieving the end TB by 2030 target. In Zambia, factors associated with treatment outcomes in children have not been extensively studied. Addressing this knowledge gap provides evidence for informed decisions and the development of interventions that would help achieve a 90% success rate. This study, therefore, set out to evaluate childhood tuberculosis outcomes and factors associated with unsuccessful treatment outcomes in selected public Hospitals in Lusaka, Zambia from 2015 to 2019.

Methods

Study design and population

The study employed a retrospective cross-sectional analysis of data from the TB registers on children aged 0 to 14 years who were diagnosed and treated for TB between 1st January 2015 and

31st December 2019. The study was conducted in all Public Level 1 Hospitals and University Teaching Hospitals within the urban City of Lusaka, Zambia. This comprised 6 level 1 Hospitals (Chawama, Chelstone, Chipata, Chilenje, Kanyama, and Matero) and 2 University Teaching Hospitals (Levi Mwanawasa University Teaching Hospital (LMUTH) and University Teaching Hospital (UTH)). These facilities conduct clinical and bacteriological diagnosis of TB in children, as they are equipped with laboratory and radiological tests as well as specialized medical personnel and act as referral Hospitals for all clinics in the city of Lusaka.

Inclusion Criteria

The study included all children aged 0 to 14 years old, diagnosed (bacteriologically or clinically), and treated with TB from the 8 public health facilities.

Exclusion Criteria

The study excluded children with missing and incomplete information in the TB registers.

Sampling and Sample Size

The study sample included all children aged 0 – 14 years who were diagnosed and treated for TB between January 2015 and December 2019 and met the selection criteria. This gave a total of 2,531 participants.

Variables and Data Extraction Procedures

Data was extracted from TB registers from all 8 Hospitals. The data extraction process was conducted from 30th November 2020 to 9th February 2021. The TB registers contained the records of all children diagnosed with TB and eligible for TB treatment, including those diagnosed with RR-TB or MDR-TB.

The data was retrieved from the TB registers using standardized extraction checklist forms that included socio-demographic characteristics (age, sex, and health facility), history of previous treatment, laboratory, and radiographic testing, HIV test results, category of TB, treatment category, and TB treatment outcomes. The diagnosis of TB was based on bacteriological and/or clinical confirmation as described in the WHO/NTP guidelines (14).

Definition of treatment outcomes

Data on treatment outcomes was retrieved from the TB registers based on the standard WHO definitions of TB treatment outcomes as follows (15); *Cured* – A pulmonary TB patient with bacteriologically confirmed TB at the beginning of treatment who was smear- or culture-negative in the last month of treatment and on at least one previous occasion; *Treatment completed* – A TB patient who completed treatment without evidence of failure BUT with no record to show that sputum smear or culture results in the last month of treatment and on at least one previous occasion were negative, either because tests were not done or because results are unavailable; *Treatment failure* – A TB patient whose sputum smear or culture is positive at month five or later during treatment; *Died* – A TB patient who dies for any reason before starting or during the course of treatment; *Lost to follow up* – A TB patient who did not start treatment or whose treatment was interrupted for two consecutive months or more; *Not evaluated* – A TB patient for whom no treatment outcome is assigned. This includes cases ‘transferred out’ to another treatment unit as well as cases for whom the treatment outcome is unknown to the reporting unit.

Furthermore, the final treatment outcome was dichotomized into successful treatment outcome = *cured* or *treatment completed* (15) and unsuccessful treatment outcome = *died, loss to follow-up* or *treatment failure*.

Data Analysis

The data were analyzed using STATA version 17.0 software. Descriptive statistics such as frequencies and percentages were used to summarize the study variables. The proportion of bacteriologically confirmed TB was calculated by taking the number of children who had positive bacteriological results and dividing it by the number of children who underwent bacteriological TB testing as the denominator. This proportion was then compared across demographic and clinical characteristics variables using the Chi-square and Fisher's exact tests.

To determine factors associated with unsuccessful treatment outcomes, logistic regression was used. The outcome variable was unsuccessful treatment outcome, a binary categorical variable which was coded =1 if unsuccessful treatment outcome, otherwise = 0. Logistic regression was performed at adjusted and unadjusted levels. Machine-lead stepwise and investigator-lead stepwise logistic regressions were used for adjusted analysis. Robust standard errors were used when taking care of clustering by health facility. The pseudo-R^2 and Bayesian Information Criteria (BIC) were used to choose the best-fit model, of which the investigator lead stepwise regression was settled for, as the best-fit model. All forms of analysis in this study were done at a 95% confidence interval.

Ethical Consideration

Ethical clearance was obtained from the University of Zambia Biomedical Research and Ethics Committee (UNZABREC) and the National Health Research Authority (NHRA) in Zambia (REF.No.1094-2020). The need to obtain informed consent from all subjects and/or their legal guardian(s) was waived by the University of Zambia Biomedical Research and Ethics Committee (UNZABREC), as the study was based on secondary data. To maintain confidentiality, names or

other identifiers of study participants were not extracted from the TB registers. All methods and procedures during this study were performed in accordance with relevant guidelines and regulations (such as the Declaration of Helsinki).

Results

Description of the study population

A total of 2,531 children's records were extracted from the TB registers as children who were treated for TB from 2015 to 2019. Out of the 2,531 children, 507 underwent microbiological testing for TB, of whom 279 had positive test results and were treated for TB following a bacteriological confirmation for TB. On the other hand, 228 children who had negative bacteriological test results and 2,024 children who did not undergo bacteriological testing for TB were treated for TB based on clinical diagnosis.

Out of the total 2,531 children who were treated for TB, only 1,495 had their treatment outcomes evaluated of whom 1,419 had a successful treatment outcome while 76 had an unsuccessful treatment outcome **(Fig 1).**

Socio-demographic and clinical characteristics of participants

Overall, out of 2,531 children who were treated for TB, 52% (1,319/2,531) were aged 0 and 4 years. There were more male children who were treated for TB 53% (1,328/2,531). UTH had the highest number of children treated for TB in the 5 years 36.1% (915/2,531) followed by Matero level 1 Hospital 21.8% (553/2,531) and Kanyama level 1 Hospital 12.7% (321/2,531) **(Table 1).**

The most common type of TB was pulmonary TB 75% (1,893/2,531). About 55.7% (1,410/2,531) of children who were treated for TB were HIV-negative. The majority of the patients 97.1% (2,454/2,531) were new patients and the initial regimen with first-line medication was used in nearly all patients 98.6% (2,495/2,531) **(Table 1).**

Table 1: Socio-demographic characteristics of children treated for TB in Lusaka (2015-2019)

Characteristics	Frequency (n) N = 2,531	Percentage (%)
**Socio-demographic characteristics of participants**		
**Age**		
0 – 4 years	1,323	52.3
5 – 14 years	1,205	47.7
**Sex**		
Female	1,196	47.4
Male	1,328	52.6
**Health facility**		
Chawama	302	11.9
Chelstone	92	3.6
Chilenje	110	4.4
Chipata	113	4.5
Kanyama	321	12.7
LMUTH	125	4.9
Matero	553	21.8
UTH	915	36.1
**Clinical characteristics of participants**		
**Type of TB**		
Pulmonary	1,893	75
Extrapulmonary	631	25
**HIV Status**		
Negative	1,410	55.7
Positive	1,037	41
Unknown	84	3.3
**Patient Type**		
New Patient	2,454	97.1
Retreatment	73	2.9
**Treatment Type**		
Initial regimen with first-line drug	2,495	98.6
Retreatment regimen with first-line drug	21	0.8
Second-line treatment drug	14	0.6

UTH – University Teaching Hospital, LMUTH – Levy Mwanawasa University Teaching Hospital, TB – Tuberculosis, HIV – Human Immune Virus, N – Sample, n – Frequency, % - Percentage, Retreatment - (treatment after failure, treatment after loss to follow up, relapse and transferred in).

Diagnostic confirmation of TB among children treated for TB

Out of the total 2,531, bacteriological tests for TB were performed on 20.0% (507/2531) of children, of whom, 55% (279/507) had a positive test result. The proportions of children who had positive test results for TB varied significantly between children under 5 years of age, 31.2% (57/183), and children over 5 years of age, 68.4% (221/323).

There was a statistically significantly higher proportion of children with pulmonary TB 60.1% (265/441) who had a positive bacteriological test result compared to children with extrapulmonary TB 21.9% (14/64). The proportion of children who had a positive bacteriological test result for TB was significantly higher in retreatment patients 83.3% (15/18) compared to new patients 54.0% (264/489). The proportion of children who had a positive bacteriological test result did not differ significantly by HIV status or sex of the participants ($p > 0.05$).

Overall, only 11% (279/2531) of children received TB treatment after a bacteriological confirmation of TB, while the remaining 89% (2,252/2,531) received TB treatment based on clinical diagnosis. Children who were treated for TB clinically comprised of children who had negative bacteriological results and those who did not undergo bacteriological testing (**Table 2).**

Table 2: Diagnostic confirmation of TB among children treated for TB, stratified by demographic and clinical characteristics (2015 - 2019)

Characteristic	Underwent bacteriological tests		Total	P value	All children treated for TB		Total	P value
	Positive n (%)	Negative n (%)			Bacteriological diagnosis n (%)	Clinical diagnosis n (%)		
**Overall**	279 (55.0)	228 (45.0)	507		279 (11.0)	2,252 (89.0)	2,531	
**Age**								
0 - 4 years	57 (31.2)	126 (68.8)	183	<0.001 C	57 (4.3)	1,266 (95.7)	1,323	<0.001 C
5 - 14 years	221 (68.4)	102 (31.6)	323		221 (18.3)	984 (81.7)	1,205	
**Sex**								
Female	141 (57.1)	106 (42.9)	247	0.365 C	141 (11.8)	1,055 (88.25)	1,196	0.263 C
Male	138 (53.1)	122 (46.9)	260		138 (10.4)	1,190 (89.6)	1,328	
**Infection Site**								
Pulmonary	265 (60.1)	176 (39.9)	441	<0.001 C	265 (14.0)	1,628 (86.0)	1,893	<0.001 C
Extrapulmonary	14 (21.9)	50 (78.1)	64		14 (2.2)	617 (97.8)	631	
**Patient Type**								
New	264 (54.0)	225 (46.0)	489	0.014 C	264 (10.8)	2,188 (89.2)	2,452	0.003 C
Relapse	15 (83.3)	3 (16.7)	18		15 (22.4)	52 (77.6)	67	
**HIV Status**								
Negative	165 (57.3)	123 (42.7)	288	0.399 E	165 (11.7)	1,245 (88.3)	1,410	0.208 C
Positive	109 (51.7)	102 (48.3)	211		109 (10.5)	928 (89.5)	1,037	
Unknown	5 (62.5)	3 (37.5)	8		5 (5.9)	79 (94.1)	84	

C - Chi-square test, E - Fishers exact C

Treatment Outcomes of Children Diagnosed and Managed for TB

The proportion of Children that had a cured treatment outcome was lowest 4.0 % (33/815) in 2018 and highest 12.6 % (34/269) in 2016. The proportion of patients who completed treatment without evidence of failure or cure was highest 66.8% (155/232) in 2015 and lowest 45.4 % (370/815) in 2018. The highest proportion of deaths 3% (8/269) was observed in 2016 and the lowest 1 % (6/629) was observed in 2017. On the other hand, treatment failure among children diagnosed with TB was very low throughout the five years and varied from 0 to 1.1 %. Loss to follow-up among children on TB treatment remained very low during the five years; the highest proportion of 2.0 %

(12/586) was recorded in 2019. The proportion of children with TB for whom no treatment outcome was assigned (not evaluated) increased over the years from 21.1 % (49/232) in 2015 to 48.5 % (395/815) in 2018 and dropped in 2019 to 32.8 % (192/586).

Most of the children at Chawama 75.2% (227/302), Chelstone 51.1% (47/92), Chilenje 78.2% (86/110), Chipata 55.7% (63/113), Kanyama 73.2% (235/321) and Matero 57.2% (316/553) had completed the treatment. However, close to 3 quarters of children from each of the two tertiary Hospitals, LMUTH 75.2% (94/125) and UTH 70.6% (646/915) did not have their outcomes evaluated.

The overall treatment outcome pattern showed that, out of the total cases of Children who had TB, 6.3% (160/2,531) were cured, 49.7% (1,259 (49.7%) were treatment completed, 1.8% (46/2,531) died, 0.9% (22/2,531) were lost to follow up, 40.9% (1,036/2,351) were not evaluated and the remaining 0.3% (8/2,531) had failed treatment.

After dropping the children that were not evaluated, treatment outcomes were categorized into successful (cured + treatment complete) and unsuccessful (died + loss to follow up + treatment failure). The highest proportion of unsuccessful treatment outcomes 8.1% (32/394) was recorded in 2019 and the lowest 2.2% (4/183) was recorded in 2015. Levy Mwanawasa University Teaching Hospital had the highest proportion 22.6% (7/31) of unsuccessful treatment, followed by Chelstone health facility 15.1% (11/73). University Teaching Hospital had the lowest proportion of unsuccessful treatment outcomes 1.9% (5/269) followed by Matero 3.2% (11/342). Seven percent of children who had extrapulmonary TB had an unsuccessful treatment outcome while only 4.4% of children with pulmonary TB had an unsuccessful treatment outcome.

The pooled estimates reviewed that 5.1% (76/1,495) clients had unsuccessful treatment outcomes while 94.9% (1,419/1,495) clients had successful treatment outcomes **(Table 3).**

Table 3: Treatment outcomes of childhood tuberculosis (2015-2019)

Variable	Category of treatment outcome n (%)							Overall treatment outcome n (%)		
	Cured	T C	Died	LTFU	TF	NE	Total	ST	UT	Total
**Overall total**	160 (6.3)	1259 (49.7)	46 (1.8)	22 (0.9)	8 (0.3)	1036 (40.9)	2531	1419 (94.9)	76 (5.1)	1495
**Years**										
2015	24 (10.3)	155 (66.8)	3 (1.3)	1 (0.4)	0 (0)	49 (21.1)	232	179 (97.8)	4 (2.2)	183
2016	34 (12.6)	124 (46.1)	8 (3.0)	1 (0.4)	3 (1.1)	99 (36.8)	269	158 (92.9)	12 (7.1)	170
2017	27 (4.3)	290 (46.1)	6 (1.0)	4 (0.6)	1 (0.2)	301 (47.9)	629	317 (96.6)	11 (3.4)	328
2018	33 (4.0)	370 (45.4)	13 (1.6)	4 (0.5)	0 (0)	395 (48.5)	815	403 (96.0)	17 (4.0)	420
2019	42 (7.2)	320 (54.6)	16 (2.7)	12 (2.0)	4 (0.7)	192 (32.8)	586	362 (91.9)	32 (8.1)	394
**Health Facility**										
Level 1 Hospitals										
Chawama	36 (11.9)	227 (75.2)	10 (3.3)	5 (1.7)	4 (1.3)	20 (6.6)	302	263 (93.1)	19 (6.7)	282
Chelstone	15 (16.3)	47 (51.1)	4 (4.4)	6 (6.5)	1 (1.1)	19 (20.6)	92	62 (84.9)	11 (15.1)	73
Chilenje	8 (7.3)	86 (78.2)	4 (3.6)	0 (0.0)	0 (0.0)	12 (10.9)	110	94 (95.9)	4 (4.1)	98
Chipata	32 (28.3)	63 (55.7)	5 (4.4)	0 (0.0)	1 (0.9)	12 (10.6)	113	95 (94.1)	6 (5.9)	101
Kanyama	51 (15.9)	235 (73.2)	9 (2.8)	2 (0.6)	2 (0.6)	22 (6.8)	321	286 (95.7)	13 (4.3)	299
Matero	15 (2.7)	316 (57.2)	5 (0.9)	6 (1.1)	0 (0.0)	211 (38.2)	553	331 (96.8)	11 (3.2)	342
Tertiary Hospitals										
LMUTH	3 (2.4)	21 (16.8)	5 (4.0)	2 (1.6)	0 (0.0)	94 (75.2)	125	24 (77.4)	7 (22.6)	31
UTH	0 (0.0)	264 (28.8)	4 (0.4)	1 (0.1)	0 (0.0)	646 (70.6)	915	264 (98.1)	5 (1.9)	269
**Type of TB**										
Extra Pulmonary	19 (3.0)	339 (53.7)	14 (2.2)	9 (1.4)	4 (0.6)	246 (39.0)	631	358 (93.0)	27 (7.0)	385
Pulmonary	139 (7.3)	919 (48.6)	32 (1.7)	13 (0.7)	4 (0.2)	786 (48.6)	1893	1058 (95.6)	49 (4.4)	1107

TC - Treatment completed, LTFU - Loss to follow up, TF - Treatment failure, NE - Not evaluated, ST - Successful treatment, UT - Unsuccessful treatment, n - frequency, % - percentage, LMUTH - Levy Mwanawasa University Teaching Hospital, UTH - University Teaching Hospital, TB - Tuberculosis. Successful - (cured + treatment complete), Unsuccessful - (died + loss to follow up + treatment failure)

Factors associated with unsuccessful treatment outcomes in children <15 years

From the univariate analysis, age, sex, type of TB, HIV status, treatment history, and diagnostic type were all not significantly associated with the unsuccessful treatment. In multivariable logistic regression, children with extrapulmonary TB were 1.64 times more likely than children with

pulmonary TB to have unsuccessful treatment outcomes (AOR 1.64; 95 % CI: 1.02 – 2.62) and this was statistically significant **(Table 4).**

Table 4: Socio-demographic and clinical factors associated with unsuccessful treatment outcome

Variable	Unsuccessful outcome n (%) N=76	Unadjusted		Adjusted	
		OR (95% CI)	P-value	OR (95% CI)	P-value
**Age group**					
0 – 4 years	43 (5.9)	1		1	
5 – 9 years	19 (4.7)	0.79 (0.58, 1.08)	0.139	0.74 (0.52, 1.07)	0.109
5 – 14 years	14 (3.9)	0.65 (0.33, 1.29)	0.223	0.62 (0.32, 1.20)	0.157
**Sex**					
Female	32 (4.8)	1		1	
Male	44 (5.8)	1.34 (0.84, 2.12)	0.217	1.34 (0.82, 2.18)	0.238
**Types of TB**					
Pulmonary	49 (4.4)	1		1	
Extra Pulmonary	27 (7.0)	1.63 (0.97, 2.73)	0.063	1.64 (1.02, 2.62)	0.042*
**HIV Status**					
Negative	40 (4.6)	1		1	
Positive	34 (5.7)	1.28 (0.62, 2.65)	0.512	1.37 (0.67, 2.78)	0.382
Unknown	2 (8.0)	1.82 (0.43, 7.73)	0.416	1.91 (0.44, 8.22)	0.382
**Treatment History**					
New Patient	73 (5.1)	1			
Retreatment	3 (5.9)	1.17 (0.36, 3.76)	0.790	-	-
**Diagnostic Type**					
Clinical	66 (5.1)	1		1	
Bacteriological	10 (5.2)	1.02 (0.42, 2.51)	0.960	1.43 (0.52, 3.90)	0.484

*TB Tuberculosis, HIV Human immune virus, CI Confidence interval, * Statistically significant at 5% significance level, OR Odds ratio, % Percentage, n Frequency, N Sample*

DISCUSSION

The current study provided valuable insights into childhood TB outcomes and factors associated with unsuccessful treatment outcome in selected public Hospitals in Lusaka, Zambia from 2015 to 2019. The findings revealed that TB was most common in children under the age of five, who were males and were HIV-negative. Pulmonary TB was the most common type of TB. Children aged 5 to 14 years, pulmonary TB, and relapse patients had a significantly higher proportion of bacteriologically confirmed tuberculosis. A high proportion of children who were treated for TB did not have their treatment outcomes evaluated. After the not-evaluated children were dropped, the successful TB treatment outcome fell above the WHO recommended threshold of 90, as the proportion of unsuccessful treatment outcome was relatively low. Extrapulmonary TB was significantly associated with unsuccessful treatment outcomes in children.

Pulmonary TB (75%) was found to be the most common type of TB in children, which is consistent with earlier studies (16,17). Extrapulmonary TB accounted for roughly a quarter of all children who had TB, which is consistent with findings from Zimbabwe (18), Ghana (19), and Pakistan (20). Other studies, however, found a higher proportion (33 % to 56 %) of extrapulmonary TB (16,21–23).

The proportion of bacteriologically confirmed TB among children who underwent bacteriological testing from 2015 to 2019 was 55%. This is substantially lower compared to findings from a Brazilian study (69%) (17). On the other hand, the proportion of bacteriologically confirmed TB among all children who were treated for TB from 2015 to 2019 was relatively low (11%). These findings are consistent with previous studies elsewhere (24). However, another study done in

Nigeria showed a higher proportion of bacteriologically confirmed TB among all children who children being treated for TB (28%) (25).

The low proportion of bacteriologically confirmed TB among all children who were treated for TB reported in the current study might be attributed to the fact that bacteriological testing for TB was only performed on 507 children out of the total 2,531 children who were treated for TB from 2015 to 2019. This meant that the majority (89%) of children were treated for TB based on clinical diagnosis which was a combination of children who had negative bacteriological test results and those who did not undergo bacteriological testing but might have been subjected to other means of diagnosis such as radiographs.

During the five years (2015-2019), the death outcome in children with tuberculosis was low, ranging from 1% to 3%, which was consistent with other studies (16,28). One of the primary problems in estimating the proportion of children who died from TB was that the exact causes of death were not recorded in the TB registers. As a result, the observed rates may be exaggerated if some deaths were caused by factors unrelated to TB treatment.

Treatment failure among children with TB was uncommon over the five years, and this finding was within the range (0% - 1%) which was similar to what has been reported in other studies (24). Furthermore, the proportion of children who were lost to follow-up on TB treatment remained very low (0.4 % - 2.1 %). This was lower than what was reported in other studies in Ethiopia (16,24).

A high proportion of children with a not-evaluated treatment outcome (40.9%) was observed over the five years. In contrast to the results of this study, other studies reported a much lower rate of not-evaluated treatment outcomes (0.1% - 22.5%) (29–31).

One of the potential reasons for having a high proportion of cases with not-evaluated treatment outcomes could have been transfers that happen between higher and lower-level health facilities. In the continuum of care, patients are diagnosed from higher-level health facilities that have the necessary equipment and human resources but later get transferred to lower-level health facilities that are near the patients' homes for continuation of treatment. This has been demonstrated in literature in a study that was done in Ethiopia, which found that most children with not-evaluated treatment outcomes were transferred (31). This was also evident in the current study as the results reviewed that over 70% of children from each of the two tertiary Hospitals (LMUTH and UTH) (Highest level facilities in Zambia) had not-evaluated treatment outcomes as compared to the six-level 1st Hospitals where the proportion of children with not-evaluated treatment outcomes was 6% to 20% with one facility reporting 38%.

This referral system between higher and lower-level health facilities often results in a break in follow-up, making it difficult to track the progress and the success of treatment outcomes in children. This also raises questions about the guarantee of continuity of care for these children.

Additionally, apart from transfers, poor documentation, and poor follow-up systems could have also contributed to treatment outcomes not being evaluated. Without proper evaluation, it becomes difficult to determine the effectiveness and success rates of the treatments being provided.

After dropping the children whose treatment outcomes were not evaluated, the unsuccessful treatment outcome ranged between 2.2% to 8.1% (2015 to 2019) with a pooled estimate of 5.1%, and the successful treatment outcome ranged between 91.9% to 97.8% (2015 to 2019) with a pooled estimate of 94.9%. This showed that the successful treatment rate met the WHO-mandated 90% treatment success threshold. The not-evaluated children were dropped to avoid

overestimating or underestimating successful and unsuccessful treatment outcomes. Our results were comparable to studies that reported similar successful treatment outcomes of 94.2% (Pakistan) (32), and 93.1% (Botswana) (33). However, other studies have reported lower successful treatment outcomes 88.6% (Ethiopia) (34), 85.4% (Pakistan) (35), 83 % (Nigeria) (36), and 58.4% (Nigeria) (37).

Regression analysis revealed that extrapulmonary TB had a higher risk of unsuccessful treatment outcomes. Other studies have come to the same conclusion (21,38). The study found no association between age, sex, HIV status, treatment history, diagnostic type, and TB unsuccessful treatment outcome. Similar results have been reported in the past by some studies (20). But other studies reported that age (10,11), sex (39), HIV status (10,40,41), treatment history, and diagnostic method (39) were associated with unsuccessful treatment outcomes in children with TB. The inconclusiveness of our findings could again be because the sample size was reduced at regression analysis as the not-evaluated children could not be exclusively classified as successful or unsuccessful outcomes to reduce bias in findings of factors associated with unsuccessful treatment outcomes, hence were excluded from the regression analysis.

The strengths that were in this study include the fact that the sample included all children treated for TB between 2015 and 2019 who met the study's selection criteria, which gave the study a big sample size. However, some limitations were also noted. Documentation in the TB registers was inadequate, there was missing data (challenges of keeping records in health facilities). This might have resulted in under or overestimation of certain TB treatment outcomes. Furthermore, there was a limitation in the number of clinical and social-demographic factors that could be investigated at regression analysis as not so many variables are captured in TB registers. The large number of not-evaluated children that was observed could have also led to overestimation or underestimation of

unsuccessful or successful treatment outcomes since these cases were dropped as they could not be classified as successful or unsuccessful treatment outcomes.

CONCLUSION

Pulmonary TB was the most common type of TB in children. The majority of children who were treated for TB from 2015 to 2019 were based on clinical diagnosis. Bacteriologically confirmed TB among children who were tested for TB was low. After removing children with not-evaluated TB treatment outcomes, the treatment success rate was above the WHO's goal of 90% for the period 2015 -2019.

Children under the age of five should be targeted as a high-risk group in strategies to eliminate childhood TB/improve TB treatment success rates with a primary focus on children with extrapulmonary TB. Future studies should explore the possibility of tracking the transferred (not-evaluated) children for better estimations of TB treatment outcomes as tertiary Hospitals had a very high number of children whose outcomes were not evaluated.

Acknowledgments

The authors are grateful to The University of Zambia, School of Public Health, Department of Epidemiology and Biostatistics. We are also grateful to the Ministry of Health, Health facility management from all 8 facilities where data was collected and all health workers operating from the TB sections in the 8 health facilities that were included in this study.

References

1. World Health Organisation. Global Tuberculosis report 2022 [Internet]. Geneva; 2022. Available from: http://apps.who.int/bookorders.
2. Tuberculosis [Internet]. [cited 2023 Jan 12]. Available from: https://www.who.int/news-room/fact-sheets/detail/tuberculosis
3. World Health Organisation. GLOBAL TUBERCULOSIS REPORT 2021 [Internet]. Geneva: 2021. 2021. Available from: http://apps.who.int/bookorders.
4. Palmer AL. Advances in Pediatric Tuberculosis [Internet]. Vol. 13, Journal of Pediatric Infectious Diseases. Georg Thieme Verlag; 2018 [cited 2020 May 12]. p. 89–90. Available from: http://www.thieme-connect.de/DOI/DOI?10.1055/s-0038-1639600
5. Thomas TA. Tuberculosis in Children. Vol. 64, Pediatric Clinics of North America. W.B. Saunders; 2017. p. 893–909.
6. World Health Organization. Framework for implementing the "End TB Strategy" in the African Region 2016-2020. [Internet]. 2017. Available from: http://apps.who.int/bookorders.
7. World Health Organization. The end TB strategy. 2015;
8. World Health Organization. Roadmap towards ending TB in children and adolescents. WHO. 2018;
9. Matucci T, Galli L, de Martino M, Chiappini E. Treating children with tuberculosis: new weapons for an old enemy. https://doi.org/101080/1120009X20191598039 [Internet]. 2019 [cited 2023 Jun 30];31(5):227–45. Available from: https://www.tandfonline.com/doi/abs/10.1080/1120009X.2019.1598039
10. Mirutse G, Fang M, Kahsay AB, Ma X. Epidemiology of childhood tuberculosis and factors associated with unsuccessful treatment outcomes in Tigray, Ethiopia: A ten-year retrospective cross sectional study. BMC Public Health. 2019 Oct 24;19(1).
11. Tilahun G, Gebre-Selassie S. Treatment outcomes of childhood tuberculosis in Addis Ababa: A five-year retrospective analysis. BMC Public Health [Internet]. 2016 Jul 21 [cited 2020 May 12];16(1):612. Available from: http://bmcpublichealth.biomedcentral.com/articles/10.1186/s12889-016-3193-8
12. Jenkins HE, Yuen CM, Rodriguez CA, Nathavitharana RR, McLaughlin MM, Donald P, et al. Mortality in children diagnosed with tuberculosis: a systematic review and meta-analysis. Lancet Infect Dis. 2017 Mar 1;17(3):285–95.
13. Joshi S. A study of treatment outcome of paediatric tuberculosis patients in an urban city of central India. Int J Community Med Public Health. 2018 Mar 23;5(4):1503.
14. World Health Organization. Definitions and reporting framework for tuberculosis - 2013 revision. 2013.
15. Linh NN, Viney K, Gegia M, Falzon D, Glaziou P, Floyd K, et al. World Health Organization treatment outcome definitions for tuberculosis: 2021 update. European Respiratory Journal [Internet]. 2021 Aug 1 [cited 2023 Jul 1];58(2). Available from: https://erj.ersjournals.com/content/58/2/2100804
16. Ramos JM, Pérez-Butragueño M, Tesfamariam A, Reyes F, Tiziano G, Endirays J, et al. Comparing tuberculosis in children aged under 5 versus 5 to 14 years old in a rural hospital in southern Ethiopia: an 18-year retrospective cross-sectional study. BMC Public Health 2019 19:1 [Internet]. 2019 Jul 2 [cited 2021 Jul 7];19(1):1–9. Available from: https://bmcpublichealth.biomedcentral.com/articles/10.1186/s12889-019-7206-2

17. Carvalho ACC, da Silva Martins P, Cardoso CAA, Miceli AL, Martire T, Sant'Anna M de FBP, et al. Pediatric tuberculosis in the metropolitan area of Rio de Janeiro. International Journal of Infectious Diseases. 2020 Sep 1;98:299–304.
18. Nzombe P, Satyanarayana S, Tweya H, Timire C, Charambira K, Ncube RT, et al. Declining Trends in Childhood TB Notifications and Profile of Notified Patients in the City of Harare, Zimbabwe, from 2009 to 2018. J Trop Med. 2020;2020.
19. Ohene SA, Fordah S, Boni P. Childhood tuberculosis and treatment outcomes in Accra: A retrospective analysis. BMC Infect Dis. 2019 Aug 28;19(1).
20. Brooks MB, Malik A, Khan S, Ahmed JF, Siddiqui S, Jaswal M, et al. Predictors of unsuccessful tuberculosis treatment outcomes in children from a prospective cohort study in Pakistan. J Glob Health [Internet]. 2021 [cited 2021 Aug 3];11:1–8. Available from: /pmc/articles/PMC7916443/
21. Abdullah A, Ahmad N, Atif M, Khan S, Wahid A, Ahmad I, et al. Treatment Outcomes of Childhood Tuberculosis in Three Districts of Balochistan, Pakistan: Findings from a Retrospective Cohort Study. J Trop Pediatr [Internet]. 2020 Jul 10 [cited 2021 Jul 28]; Available from: https://academic.oup.com/tropej/advance-article/doi/10.1093/tropej/fmaa042/5869556
22. Ogbudebe C, Adepoju V, Ekerete-Udofia C, Abu E, Egesemba G, Chukwueme N. Childhood Tuberculosis in Nigeria: Disease Presentation and Treatment Outcomes. Health Serv Insights. 2018;11.
23. Khantee P, Laoprasopwattana K. Comparing Treatment Outcomes between Confirmed and Non-Confirmed Childhood TB Cases: Study from a Major Tertiary Center in Thailand. J Trop Pediatr [Internet]. 2021 [cited 2021 Aug 8];67(1). Available from: https://academic.oup.com/tropej/article/67/1/fmaa125/6075108
24. Abdullah A, Ahmad N, Atif M, Khan S, Wahid A, Ahmad I, et al. Treatment Outcomes of Childhood Tuberculosis in Three Districts of Balochistan, Pakistan: Findings from a Retrospective Cohort Study. J Trop Pediatr [Internet]. 2021 Jul 2 [cited 2021 Aug 7];67(3). Available from: https://academic.oup.com/tropej/article/67/3/fmaa042/5869556
25. Ogbudebe CL, Adepoju V, Ekerete-Udofia C, Abu E, Egesemba G, Chukwueme N, et al. Childhood Tuberculosis in Nigeria: Disease Presentation and Treatment Outcomes. Health Serv Insights [Internet]. 2018 [cited 2023 Jun 30];11. Available from: https://pubmed.ncbi.nlm.nih.gov/29511357/
26. Dunn JJ, Starke JR, Revell PA. Laboratory diagnosis of mycobacterium tuberculosis infection and disease in children. J Clin Microbiol. 2016 Jun 1;54(6):1434–41.
27. Gutiérrez-González LH, Juárez E, Carranza C, Carreto-Binaghi LE, Alejandre A, Cabello-Gutiérrrez C, et al. Immunological Aspects of Diagnosis and Management of Childhood Tuberculosis. Infect Drug Resist [Internet]. 2021 [cited 2021 Nov 22];14:929. Available from: /pmc/articles/PMC7955028/
28. Laghari M, Sulaiman SAS, Khan AH, Memon N. A prospective study of socio-demographic, clinical characteristics and treatment outcomes of children with tuberculosis in Sindh, Pakistan. BMC Infect Dis [Internet]. 2019 Jan 24 [cited 2020 Aug 22];19(1):1–11. Available from: https://link.springer.com/articles/10.1186/s12879-019-3702-3
29. Abdullah A, Ahmad N, Atif M, Khan S, Wahid A, Ahmad I, et al. Treatment Outcomes of Childhood Tuberculosis in Three Districts of Balochistan, Pakistan: Findings from a Retrospective Cohort Study. J Trop Pediatr [Internet]. 2021 Jul 2 [cited 2021 Aug 7];67(3). Available from: https://academic.oup.com/tropej/article/67/3/fmaa042/5869556

30. Hamid M, Brooks MB, Madhani F, Ali H, Naseer MJ, Becerra M, et al. Risk factors for unsuccessful tuberculosis treatment outcomes in children. EHTESHAM HS, editor. PLoS One [Internet]. 2019 Sep 25 [cited 2020 May 12];14(9):e0222776. Available from: http://dx.plos.org/10.1371/journal.pone.0222776
31. Mirutse G, Fang M, Kahsay AB, Ma X. Epidemiology of childhood tuberculosis and factors associated with unsuccessful treatment outcomes in Tigray, Ethiopia: A ten-year retrospective cross sectional study. BMC Public Health. 2019 Oct 24;19(1).
32. Brooks MB, Malik A, Khan S, Ahmed JF, Siddiqui S, Jaswal M, et al. Predictors of unsuccessful tuberculosis treatment outcomes in children from a prospective cohort study in Pakistan. J Glob Health [Internet]. 2021 [cited 2023 Jul 2];11:1–8. Available from: /pmc/articles/PMC7916443/
33. Siamisang K, Rankgoane-Pono G, Madisa TM, Mudiayi TK, Tlhakanelo JT, Mubiri P, et al. Pediatric tuberculosis outcomes and factors associated with unfavorable treatment outcomes in Botswana, 2008–2019: a retrospective analysis. BMC Public Health [Internet]. 2022 Dec 1 [cited 2023 Jul 2];22(1):1–8. Available from: https://bmcpublichealth.biomedcentral.com/articles/10.1186/s12889-022-14477-y
34. Weldegebreal F, Teklemariam Z, Mitiku H, Tesfaye T, Abrham Roba A, Tebeje F, et al. Treatment outcome of pediatric tuberculosis in eastern Ethiopia. Front Pediatr. 2022 Aug 11;10:966237.
35. Hamid M, Brooks MB, Madhani F, Ali H, Naseer MJ, Becerra M, et al. Risk factors for unsuccessful tuberculosis treatment outcomes in children. PLoS One [Internet]. 2019 Sep 1 [cited 2023 Jul 2];14(9). Available from: /pmc/articles/PMC6760830/
36. Ogbudebe CL, Adepoju V, Ekerete-Udofia C, Abu E, Egesemba G, Chukwueme N, et al. Childhood Tuberculosis in Nigeria: Disease Presentation and Treatment Outcomes. https://doi.org/101177/1178632918757490 [Internet]. 2018 Feb 22 [cited 2023 Jul 2];11. Available from: https://journals.sagepub.com/doi/10.1177/1178632918757490
37. Alex-Hart BA, Paul NI, Alex-Hart BA, Paul NI. Pattern and Outcome of Childhood Tuberculosis Seen at the University of Port Harcourt Teaching Hospital, Nigeria. J Tuberc Res [Internet]. 2019 Sep 3 [cited 2023 Jul 2];7(3):170–83. Available from: http://www.scirp.org/journal/PaperInformation.aspx?PaperID=95501
38. Li T, Ma Y, Liu K, Shewade HD, Zhang H, Du X, et al. Childhood TB in China: notification, characteristics and risk factors for outcomes, 2010–2017. International Journal of Tuberculosis and Lung Disease. 2020 Dec 1;24(12):1285–93.
39. Hamid M, Brooks MB, Madhani F, Ali H, Naseer MJ, Becerra M, et al. Risk factors for unsuccessful tuberculosis treatment outcomes in children. EHTESHAM HS, editor. PLoS One [Internet]. 2019 Sep 25 [cited 2020 May 12];14(9):e0222776. Available from: http://dx.plos.org/10.1371/journal.pone.0222776
40. Osman M, Lee K, du Preez K, Dunbar R, Hesseling AC, Seddon JA, et al. EXCELLENT TREATMENT OUTCOMES IN CHILDREN TREATED FOR TUBERCULOSIS UNDER ROUTINE OPERATIONAL CONDITIONS IN CAPE TOWN AUTHORS. 2017;
41. Belay GM, Wubneh CA. Childhood tuberculosis treatment outcome and its association with HIV co-infection in Ethiopia: A systematic review and meta-analysis [Internet]. Vol. 48, Tropical Medicine and Health. BioMed Central Ltd.; 2020 [cited 2020 Aug 28]. Available from: /pmc/articles/PMC7027074/?report=abstract

Click here to access/download;Figure;Fig 1.docx

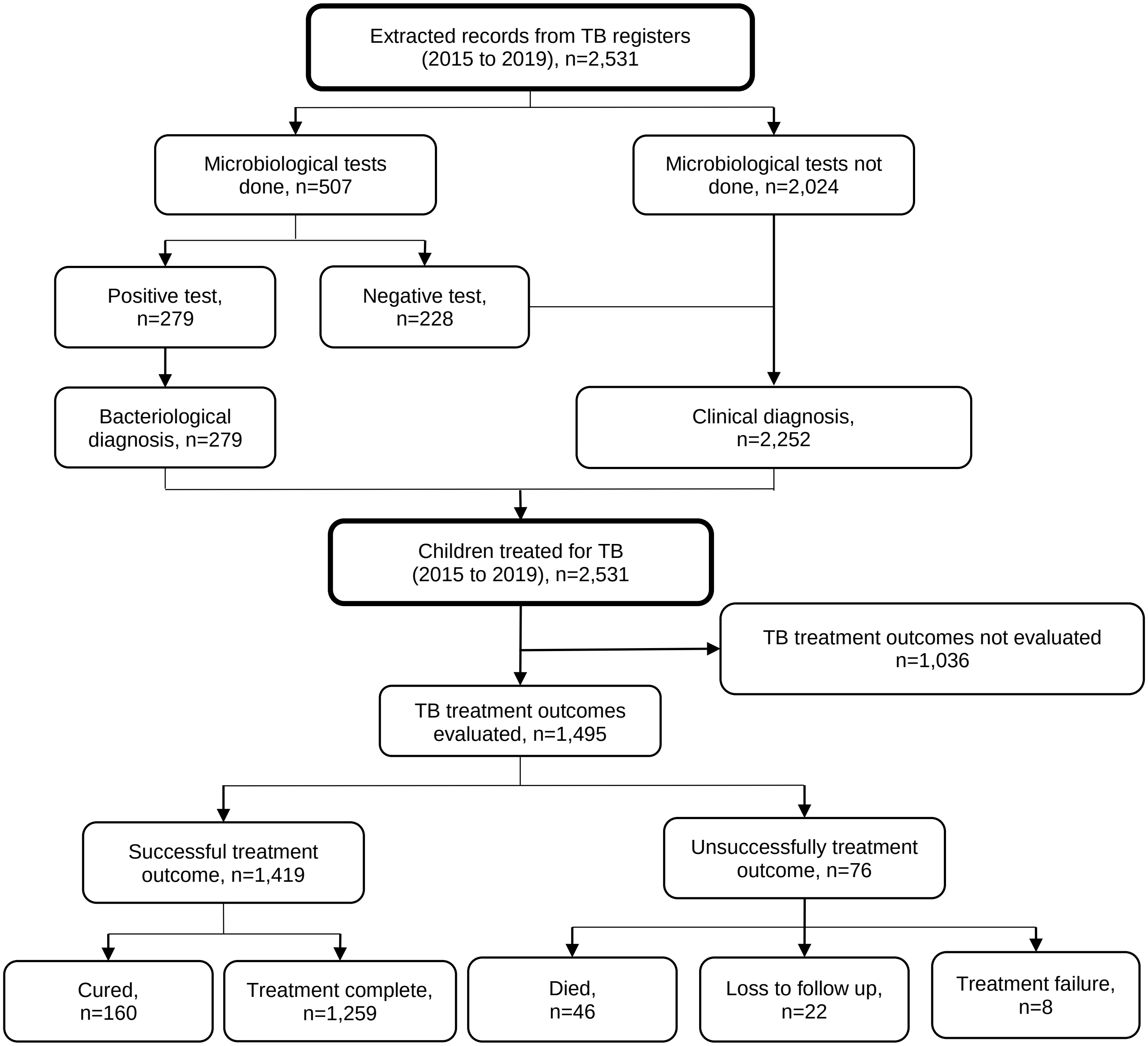

TB Tuberculosis, n Sample

Fig 1: Study flow chart

Childhood Tuberculosis Outcomes and Factors Associated with Unsuccessful Treatment Outcomes in Selected Public Hospitals of Lusaka Zambia from 2015 to 2019

Dennis Ngosa [1,2*¶], Joseph Lupenga[1¶]

1 The University of Zambia, Department of Epidemiology and Biostatistics, School of Public Health, Lusaka, Zambia

2 Analysis Department, Centre for Infectious Diseases Research in Zambia, Lusaka, Zambia

***Corresponding Author:** dennisngosam@gmail.com*

¶ These authors contributed equally to this work

Abstract

~~**Background:**~~ Treatment outcomes of tuberculosis in children are rarely evaluated. Childhood tuberculosis has been a low priority for tuberculosis programs due to difficulties in diagnosis and treatment. This study evaluated childhood tuberculosis outcomes and factors associated with unsuccessful treatment outcomes in selected public Hospitals in Lusaka, Zambia from 2015 to 2019.

~~**Methods:**~~ This was a retrospective cross-sectional study conducted in 8 public Hospitals in Lusaka. All children aged 0 – 14 years, treated with tuberculosis were included. The WHO tuberculosis treatment outcomes were grouped into successful treatment outcome (cured, treatment completed) and unsuccessful treatment outcome (death, loss to follow-up, failure). Taking unsuccessful treatment outcome as the outcome variable, unadjusted and adjusted logistic regressions were performed. All analyses were done at a 95% confidence interval.

~~**Results:**~~ Out of 2,531 participants, majority were 0 to 4 years old (52.3%), males (52.6%), HIV-negative (55.7%), and had pulmonary tuberculosis (75%). About 80% of children, were treated for TB based on clinical diagnosis. Bacteriological tests were performed on 20% of children, where 55% had positive bacteriological results. Bacteriologically confirmed TB was higher in children over 5 years (68.4%), pulmonary TB (60.1%), and retreatment (83.3%). ~~The proportion of bacteriologically confirmed tuberculosis was 11% and was significantly higher in children aged 5 to 14 years (18.3%), pulmonary tuberculosis (14.0%), and relapse patients (22.4%).~~ Overall, 6.3% of patients were cured, 49.7% completed treatment, 0.9% lost to follow-up, 1.8% died, 0.3% failed treatment and 40.9% were not evaluated. The overall unsuccessful treatment outcome was 5.1% with a successful treatment outcome of 94.1% after the not-evaluated children were dropped.

Extrapulmonary tuberculosis was associated with unsuccessful treatment outcomes (AOR 1.64; 95 % CI: 1.02 – 2.62).

~~Conclusion:~~ A high proportion of children were not evaluated for treatment outcomes. The successful treatment outcome met the World Health Organization's threshold goal of 90% after children who were not evaluated were dropped. Children with extrapulmonary tuberculosis should be targeted as a high-risk group to improve treatment outcomes.

Keywords: *Treatment outcomes, tuberculosis, Unsuccessful treatment outcomes*

Introduction

Before the coronavirus (COVID-19) pandemic, tuberculosis (TB) was the most common infectious cause of death, surpassing HIV/AIDS (1). An estimated 10.8 million new cases of the illness and over 1.6 million estimated TB deaths were recorded globally in 2021 (1). Out of these, 6 million were men, 3.4 million were women and 1.2 million were children (1).

Although TB affects adults more frequently than children, children are often seen as a specific risk group. This is because TB in children can be challenging to diagnose and treat (1–3). Furthermore, TB in children and adolescents is frequently overlooked by health providers and has been a low priority for national TB programs (4,5).

Ending the TB epidemic by 2030 is one of the Sustainable Development Goals' health targets (6) (6). Zambia is among the countries that have committed to the end TB by 2030 strategy, which includes implementing the 90-90-90 TB strategy (8). One of the three main goals is to achieve a 90 percent treatment success rate among those who have been identified as needing treatment (7).

However, the TB treatment outcome data (successful or unsuccessful treatment outcomes) for children 0–14 years are not reported in most countries (8). A systematic review on treating children with TB reported that children have been left behind and neglected as data on many aspects of the treatment of childhood TB is still scarce, and the paucity of high-quality data is concerning (9).

Previous studies have shown that the outcome of TB treatment in children varies with age, sex, HIV status, and type of TB (10,11). Several factors put children who are diagnosed with TB disease and initiated on treatment at higher risk of experiencing unsuccessful treatment outcomes, including treatment failure, death, or being lost to follow-up. Sex, age, and type of TB have been reported to be associated with unsuccessful treatment outcomes (10,12). However, other studies argued that sex, age, and type of TB were not associated with unsuccessful treatment outcomes. (13).

Monitoring treatment outcomes in children with TB and understanding the factors associated with unsuccessful treatment outcomes is critical in achieving the end TB by 2030 target. In Zambia, factors associated with treatment outcomes in children have not been extensively studied. Addressing this knowledge gap provides evidence for informed decisions and the development of interventions that would help achieve a 90% success rate. This study, therefore, set out to evaluate childhood tuberculosis outcomes and factors associated with unsuccessful treatment outcomes in selected public Hospitals in Lusaka, Zambia from 2015 to 2019.

Methods

Study design and population

The study employed a retrospective cross-sectional analysis of data from the TB registers on children aged 0 to 14 years who were diagnosed and treated for TB between 1st January 2015 and 31st December 2019. The study was conducted in all Public Level 1 Hospitals and University Teaching Hospitals within the urban City of Lusaka, Zambia. This comprised 6 level 1 Hospitals (Chawama, Chelstone, Chipata, Chilenje, Kanyama, and Matero) and 2 University Teaching Hospitals (Levi Mwanawasa University Teaching Hospital (LMUTH) and University Teaching Hospital (UTH)). These facilities conduct clinical and bacteriological diagnosis of TB in children, as they are equipped with laboratory and radiological tests as well as specialized medical personnel and act as referral Hospitals for all clinics in the city of Lusaka.

Inclusion Criteria

The study included all children aged 0 to 14 years old, diagnosed (bacteriologically or clinically), and treated with TB from the 8 public health facilities.

Exclusion Criteria

The study excluded children with missing and incomplete information in the TB registers.

Sampling and Sample Size

The study sample included all children aged 0 – 14 years who were diagnosed and treated for TB between January 2015 and December 2019 and met the selection criteria. This gave a total of 2,531 participants.

Variables and Data Extraction Procedures

Data was extracted from TB registers from all 8 Hospitals. The data extraction process was conducted from 30th November 2020 to 9th February 2021. The TB registers contained the records of all children diagnosed with TB and eligible for TB treatment, including those diagnosed with RR-TB or MDR-TB.

The data was retrieved from the TB registers using standardized extraction checklist forms that included socio-demographic characteristics (age, sex, and health facility), history of previous treatment, laboratory, and radiographic testing, HIV test results, category of TB, treatment category, and TB treatment outcomes. The diagnosis of TB was based on bacteriological and/or clinical confirmation as described in the WHO/NTP guidelines (14).

Definition of treatment outcomes

Data on treatment outcomes was retrieved from the TB registers based on the standard WHO definitions of TB treatment outcomes as follows (15); *Cured* – A pulmonary TB patient with bacteriologically confirmed TB at the beginning of treatment who was smear- or culture-negative in the last month of treatment and on at least one previous occasion; *Treatment completed* – A TB patient who completed treatment without evidence of failure BUT with no record to show that sputum smear or culture results in the last month of treatment and on at least one previous occasion were negative, either because tests were not done or because results are unavailable; *Treatment failure* – A TB patient whose sputum smear or culture is positive at month five or later during treatment; *Died* – A TB patient who dies for any reason before starting or during the course of treatment; *Lost to follow up* – A TB patient who did not start treatment or whose treatment was interrupted for two consecutive months or more; *Not evaluated* – A TB patient for whom no

treatment outcome is assigned. This includes cases ‘transferred out’ to another treatment unit as well as cases for whom the treatment outcome is unknown to the reporting unit.

Furthermore, the final treatment outcome was dichotomized into successful treatment outcome = *cured* or *treatment completed* (15) and unsuccessful treatment outcome = *died, loss to follow-up* or *treatment failure*.

Data Analysis

The data were analyzed using STATA version 17.0 software. Descriptive statistics such as frequencies and percentages were used to summarize the study variables. The proportion of bacteriologically confirmed TB was calculated by taking the number of children who had positive bacteriological results and dividing it by the number of children who underwent bacteriological TB testing as the denominator. This proportion was then compared across demographic and clinical characteristics variables using the Chi-square and Fisher’s exact tests.

To determine factors associated with unsuccessful treatment outcomes, logistic regression was used. The outcome variable was unsuccessful treatment outcome, a binary categorical variable which was coded =1 if unsuccessful treatment outcome, otherwise = 0. Logistic regression was performed at adjusted and unadjusted levels. Machine-lead stepwise and investigator-lead stepwise logistic regressions were used for adjusted analysis. Robust standard errors were used when taking care of clustering by health facility. The pseudo-R^2 and Bayesian Information Criteria (BIC) were used to choose the best-fit model, of which the investigator lead stepwise regression was settled for, as the best-fit model. All forms of analysis in this study were done at a 95% confidence interval.

Ethical Consideration

Ethical clearance was obtained from the University of Zambia Biomedical Research and Ethics Committee (UNZABREC) and the National Health Research Authority (NHRA) in Zambia (REF.No.1094-2020). The need to obtain informed consent from all subjects and/or their legal guardian(s) was waived by the University of Zambia Biomedical Research and Ethics Committee (UNZABREC), as the study was based on secondary data. To maintain confidentiality, names or other identifiers of study participants were not extracted from the TB registers. All methods and procedures during this study were performed in accordance with relevant guidelines and regulations (such as the Declaration of Helsinki).

Results

Description of the study population

A total of 2,531 children's records were extracted from the TB registers as children who were treated for TB from 2015 to 2019. Out of the 2,531 children, 507 underwent microbiological testing for TB, of whom 279 had positive test results and were treated for TB following a bacteriological confirmation for TB. On the other hand, 228 children who had negative bacteriological test results and 2,024 children who did not undergo bacteriological testing for TB were treated for TB based on clinical diagnosis.

Out of the total 2,531 children who were treated for TB, only 1,495 had their treatment outcomes evaluated of whom 1,419 had a successful treatment outcome while 76 had an unsuccessful treatment outcome **(Fig 1).**

Socio-demographic and clinical characteristics of participants

Overall, out of 2,531 children who were treated for TB, 52% (1,319/2,531) were aged 0 and 4 years. There were more male children who were treated for TB 53% (1,328/2,531). UTH had the highest number of children treated for TB in the 5 years 36.1% (915/2,531) followed by Matero level 1 Hospital 21.8% (553/2,531) and Kanyama level 1 Hospital 12.7% (321/2,531) **(Table 1).**

The most common type of TB was pulmonary TB 75% (1,893/2,531). About 55.7% (1,410/2,531) of children who were treated for TB were HIV-negative. The majority of the patients 97.1% (2,454/2,531) were new patients and the initial regimen with first-line medication was used in nearly all patients 98.6% (2,495/2,531) **(Table 1).**

Table 1: Socio-demographic characteristics of children treated for TB in Lusaka (2015-2019)

Characteristics	Frequency (n) N = 2,531	Percentage (%)
**Socio-demographic characteristics of participants**		
**Age**		
0 – 4 years	1,323	52.3
5 – 14 years	1,205	47.7
**Sex**		
Female	1,196	47.4
Male	1,328	52.6
**Health facility**		
Chawama	302	11.9
Chelstone	92	3.6
Chilenje	110	4.4
Chipata	113	4.5
Kanyama	321	12.7
LMUTH	125	4.9
Matero	553	21.8
UTH	915	36.1
**Clinical characteristics of participants**		
**Type of TB**		
Pulmonary	1,893	75
Extrapulmonary	631	25
**HIV Status**		
Negative	1,410	55.7
Positive	1,037	41
Unknown	84	3.3
**Patient Type**		
New Patient	2,454	97.1
Retreatment	73	2.9
**Treatment Type**		

Initial regimen with first-line drug	2,495	98.6
Retreatment regimen with first-line drug	21	0.8
Second-line treatment drug	14	0.6

UTH – University Teaching Hospital, LMUTH – Levy Mwanawasa University Teaching Hospital, TB – Tuberculosis, HIV – Human Immune Virus, N = Sample, n= Frequency, % = Percentage, Retreatment=(treatment after failure, treatment after loss to follow up, relapse and transferred in),

Diagnostic confirmation of TB among children treated for TB

Out of the total 2,531, bacteriological tests for TB were performed on 20.0% (507/2531) of children, of whom, 55% (279/507) had a positive test result. The proportions of children who had positive test results for TB varied significantly between children under 5 years of age, 31.2% (57/183), and children over 5 years of age, 68.4% (221/323).

There was a statistically significantly higher proportion of children with pulmonary TB 60.1% (265/441) who had a positive bacteriological test result compared to children with extrapulmonary TB 21.9% (14/64). The proportion of children who had a positive bacteriological test result for TB was significantly higher in retreatment patients 83.3% (15/18) compared to new patients 54.0% (264/489). The proportion of children who had a positive bacteriological test result did not differ significantly by HIV status or sex of the participants ($p > 0.05$).

Overall, only 11% (279/2531) of children received TB treatment after a bacteriological confirmation of TB, while the remaining 89% (2,252/2,531) received TB treatment on the basis of

clinical diagnosis. Children who were treated for TB clinically comprised of children who had negative bacteriological results and those who did not undergo bacteriological testing (**Table 2).**

Proportion of Bacteriologically Confirmed TB in children < 15 years

From 2015 to 2019, the overall proportion of bacteriologically diagnosed childhood TB was 11% (279/2,531) (95% CI; 7.2% - 16.4%). There was a statistically significant difference in bacteriologically confirmed TB proportion between children less than 5 years 4.3% (57/1,323) (95% CI; 3.2% – 5.4%) and children who were 5 years and above 18.3% (221/1,205) (95% CI; 16.2% – 20.5%). The proportion of bacteriologically confirmed TB was slightly higher in females 11.7% (141/1,196) (95% CI; 10.0% – 13.6%) as compared to males 10.4% (138/1,328) (95% CI; 8.8% – 12.0%), however, the difference was not statistically significant. Regarding the site of TB infection, the proportion of bacteriologically confirmed TB was significantly higher in children with pulmonary TB infection 14.0% (265/1,893) (95% CI; 12.4% – 15.6%) as compared to children with extrapulmonary TB infection 2.2% (14/631) (95% CI; 1.1% – 3.4%). The proportion of bacteriologically confirmed TB was significantly higher in patients who were retreated 22.4% (15/67) (95% CI; 12.4% – 32.4%) compared to new patients 10.7% (264/2,452) (95% CI; 9.5% –

~~12.0%). No significant difference in the proportion of bacteriologically confirmed TB was observed between HIV-positive children 10.5% (109/1,037) (95% CI; 8.6% – 12.4%) and HIV-negative children 11.7% (165/1,410) (95% CI; 10.0% – 13.4%) (Table 2).~~

Table 2: Diagnostic confirmation of TB among children treated for TB, stratified by demographic and clinical characteristics (2015 - 2019)

Characteristic	Underwent bacteriological tests		Total	P value	All children treated for TB		Total	P value
	Positive n (%)	Negative n (%)			Bacteriological diagnosis n (%)	Clinical diagnosis n (%)		
**Overall**	279 (55.0)	228 (45.0)	507		279 (11.0)	2,252 (89.0)	2,531	-
**Age**								-
0 - 4 years	57 (31.2)	126 (68.8)	183	<0.001 C	57 (4.3)	1,266 (95.7)	1,323	<0.001 C
5 - 14 years	221 (68.4)	102 (31.6)	323		221 (18.3)	984 (81.7)	1,205	-
**Sex**								-
Female	141 (57.1)	106 (42.9)	247	0.365 C	141 (11.8)	1,055 (88.25)	1,196	0.263 C
Male	138 (53.1)	122 (46.9)	260		138 (10.4)	1,190 (89.6)	1,328	-
**Infection Site**								-
Pulmonary	265 (60.1)	176 (39.9)	441	<0.001 C	265 (14.0)	1,628 (86.0)	1,893	<0.001 C
Extrapulmonary	14 (21.9)	50 (78.1)	64		14 (2.2)	617 (97.8)	631	-
**Patient Type**								-
New	264 (54.0)	225 (46.0)	489	0.014 C	264 (10.8)	2,188 (89.2)	2,452	0.003 C
Relapse	15 (83.3)	3 (16.7)	18		15 (22.4)	52 (77.6)	67	-
**HIV Status**								-
Negative	165 (57.3)	123 (42.7)	288	0.399 E	165 (11.7)	1,245 (88.3)	1,410	0.208 C
Positive	109 (51.7)	102 (48.3)	211		109 (10.5)	928 (89.5)	1,037	-
Unknown	5 (62.5)	3 (37.5)	8	-	5 (5.9)	79 (94.1)	84	-

C - Chi-square test, E - Fishers exact C

~~**Table 2: Bacteriologically confirmed childhood TB proportions in Lusaka (2015 - 2019)**~~

~~Characteristics~~	~~Diagnostic type~~		~~Total cases~~	~~Prevalence of Bacteriological TB % (95% CI)~~	~~Z test P-value~~
	~~Bacteriological~~	~~Clinical~~			
~~Overall~~	~~279~~	~~2,252~~	~~2,531~~	~~11.0 (7.2 – 16.4)~~	
~~Age~~					
~~Less than 5 years~~	~~57~~	~~1,266~~	~~1,323~~	~~4.3 (3.2 – 5.4)~~	~~<0.001*~~

5 – 14 years	221	984	1,205	18.3 (16.2 – 20.5)	
**Sex**					
Female	141	1,055	1,196	11.7 (10.0 – 13.6)	0.263
Male	138	1,190	1,328	10.4 (8.8 – 12.0)	
**Infection site**					
Pulmonary	265	1,628	1,893	14.0 (12.4 – 15.6)	<0.001*
Extra Pulmonary	14	617	631	2.2 (1.1 – 3.4)	
**Patient Type**					
New Patient	264	2,188	2,452	10. 7 (9.5 – 12.0)	0.018*
Retreatment	15	52	67	22.4 (12.4 – 32.4)	
**HIV status**					
Negative	165	1,245	1,410	11.7 (10.0 – 13.4)	0.208
Positive	109	928	1,037	10.5 (8.6 – 12.4)	

*Z = Z test for a proportion, * = Statistically significant at 5% significance level, CI – Confidence Interval, TB - Tuberculosis*

Treatment Outcomes of Children Diagnosed and Managed for TB

The proportion of Children that had a cured treatment outcome was lowest 4.0 % (33/815) in 2018 and highest 12.6 % (34/269) in 2016. The proportion of patients who completed treatment without evidence of failure or cure was highest 66.8% (155/232) in 2015 and lowest 45.4 % (370/815) in 2018. The highest proportion of deaths 3% (8/269) was observed in 2016 and the lowest 1 % (6/629) was observed in 2017. On the other hand, treatment failure among children diagnosed with TB was very low throughout the five years and varied from 0 to 1.1 %. Loss to follow-up among children on TB treatment remained very low during the five years; the highest proportion of 2.0 % (12/586) was recorded in 2019. The proportion of children with TB for whom no treatment outcome was assigned (not evaluated) increased over the years from 21.1 % (49/232) in 2015 to 48.5 % (395/815) in 2018 and dropped in 2019 to 32.8 % (192/586).

Most of the children at Chawama 75.2% (227/302), Chelstone 51.1% (47/92), Chilenje 78.2% (86/110), Chipata 55.7% (63/113), Kanyama 73.2% (235/321) and Matero 57.2% (316/553) had

completed the treatment. However, close to 3 quarters of children from each of the two tertiary Hospitals, LMUTH 75.2% (94/125) and UTH 70.6% (646/915) did not have their outcomes evaluated.

The overall treatment outcome pattern showed that, out of the total cases of Children who had TB, 6.3% (160/2,531) were cured, 49.7% (1,259 (49.7%) were treatment completed, 1.8% (46/2,531) died, 0.9% (22/2,531) were lost to follow up, 40.9% (1,036/2,351) were not evaluated and the remaining 0.3% (8/2,531) had failed treatment.

After dropping the children that were not evaluated, treatment outcomes were categorized into successful (cured + treatment complete) and unsuccessful (died + loss to follow up + treatment failure). The highest proportion of unsuccessful treatment outcomes 8.1% (32/394) was recorded in 2019 and the lowest 2.2% (4/183) was recorded in 2015. Levy Mwanawasa University Teaching Hospital had the highest proportion 22.6% (7/31) of unsuccessful treatment, followed by Chelstone health facility 15.1% (11/73). University Teaching Hospital had the lowest proportion of unsuccessful treatment outcomes 1.9% (5/269) followed by Matero 3.2% (11/342). Seven percent of children who had extrapulmonary TB had an unsuccessful treatment outcome while only 4.4% of children with pulmonary TB had an unsuccessful treatment outcome.

The pooled estimates reviewed that 5.1% (76/1,495) clients had unsuccessful treatment outcomes while 94.9% (1,419/1,495) clients had successful treatment outcomes **(Table 3).**

Table 3: Treatment outcomes of childhood tuberculosis (2015-2019)

Variable	Category of treatment outcome n (%)							Overall treatment outcome n (%)		
	Cured	T C	Died	LTFU	TF	NE	Total	ST	UT	Total
**Overall total**	160 (6.3)	1259 (49.7)	46 (1.8)	22 (0.9)	8 (0.3)	1036 (40.9)	2531	1419 (94.9)	76 (5.1)	1495
**Years**										

2015	24 (10.3)	155 (66.8)	3 (1.3)	1 (0.4)	0 (0)	49 (21.1)	232	179 (97.8)	4 (2.2)	183
2016	34 (12.6)	124 (46.1)	8 (3.0)	1 (0.4)	3 (1.1)	99 (36.8)	269	158 (92.9)	12 (7.1)	170
2017	27 (4.3)	290 (46.1)	6 (1.0)	4 (0.6)	1 (0.2)	301 (47.9)	629	317 (96.6)	11 (3.4)	328
2018	33 (4.0)	370 (45.4)	13 (1.6)	4 (0.5)	0 (0)	395 (48.5)	815	403 (96.0)	17 (4.0)	420
2019	42 (7.2)	320 (54.6)	16 (2.7)	12 (2.0)	4 (0.7)	192 (32.8)	586	362 (91.9)	32 (8.1)	394
**Health Facility**										
Level 1 Hospitals										
Chawama	36 (11.9)	227 (75.2)	10 (3.3)	5 (1.7)	4 (1.3)	20 (6.6)	302	263 (93.1)	19 (6.7)	282
Chelstone	15 (16.3)	47 (51.1)	4 (4.4)	6 (6.5)	1 (1.1)	19 (20.6)	92	62 (84.9)	11 (15.1)	73
Chilenje	8 (7.3)	86 (78.2)	4 (3.6)	0 (0.0)	0 (0.0)	12 (10.9)	110	94 (95.9)	4 (4.1)	98
Chipata	32 (28.3)	63 (55.7)	5 (4.4)	0 (0.0)	1 (0.9)	12 (10.6)	113	95 (94.1)	6 (5.9)	101
Kanyama	51 (15.9)	235 (73.2)	9 (2.8)	2 (0.6)	2 (0.6)	22 (6.8)	321	286 (95.7)	13 (4.3)	299
Matero	15 (2.7)	316 (57.2)	5 (0.9)	6 (1.1)	0 (0.0)	211 (38.2)	553	331 (96.8)	11 (3.2)	342
Tertiary Hospitals										
LMUTH	3 (2.4)	21 (16.8)	5 (4.0)	2 (1.6)	0 (0.0)	94 (75.2)	125	24 (77.4)	7 (22.6)	31
UTH	0 (0.0)	264 (28.8)	4 (0.4)	1 (0.1)	0 (0.0)	646 (70.6)	915	264 (98.1)	5 (1.9)	269
**Type of TB**										
Extra Pulmonary	19 (3.0)	339 (53.7)	14 (2.2)	9 (1.4)	4 (0.6)	246 (39.0)	631	358 (93.0)	27 (7.0)	385
Pulmonary	139 (7.3)	919 (48.6)	32 (1.7)	13 (0.7)	4 (0.2)	786 (48.6)	1893	1058 (95.6)	49 (4.4)	1107

TC - Treatment completed, LTFU - Loss to follow up, TF - Treatment failure, NE - Not evaluated, ST - Successful treatment, UT - Unsuccessful treatment, n - frequency, % - percentage, LMUTH – Levy Mwanawasa University Teaching Hospital, UTH – University Teaching Hospital, TB – Tuberculosis. Successful = (cured + treatment complete), Unsuccessful = (died + loss to follow up + treatment failure)

Factors associated with unsuccessful treatment outcomes in children <15 years

From the univariate analysis, age, sex, type of TB, HIV status, treatment history, and diagnostic type were all not significantly associated with the unsuccessful treatment. In multivariable logistic regression, children with extrapulmonary TB were 1.64 times more likely than children with pulmonary TB to have unsuccessful treatment outcomes (AOR 1.64; 95 % CI: 1.02 – 2.62) and this was statistically significant **(Table 4).**

Table 4: Socio-demographic and clinical factors associated with unsuccessful treatment outcome

Variable	Unsuccessful outcome n (%) N=76	Unadjusted		Adjusted	
		**OR (95% CI)**	**P-value**	**OR (95% CI)**	**P-value**
**Age group**					

0 – 4 years	43 (5.9)	1		1	
5 – 9 years	19 (4.7)	0.79 (0.58, 1.08)	0.139	0.74 (0.52, 1.07)	0.109
5 – 14 years	14 (3.9)	0.65 (0.33, 1.29)	0.223	0.62 (0.32, 1.20)	0.157
**Sex**					
Female	32 (4.8)	1		1	
Male	44 (5.8)	1.34 (0.84, 2.12)	0.217	1.34 (0.82, 2.18)	0.238
**Types of TB**					
Pulmonary	49 (4.4)	1		1	
Extra Pulmonary	27 (7.0)	1.63 (0.97, 2.73)	0.063	1.64 (1.02, 2.62)	0.042*
**HIV Status**					
Negative	40 (4.6)	1		1	
Positive	34 (5.7)	1.28 (0.62, 2.65)	0.512	1.37 (0.67, 2.78)	0.382
Unknown	2 (8.0)	1.82 (0.43, 7.73)	0.416	1.91 (0.44, 8.22)	0.382
**Treatment History**					
New Patient	73 (5.1)	1			
Retreatment	3 (5.9)	1.17 (0.36, 3.76)	0.790	-	-
**Diagnostic Type**					
Clinical	66 (5.1)	1		1	
Bacteriological	10 (5.2)	1.02 (0.42, 2.51)	0.960	1.43 (0.52, 3.90)	0.484

*TB Tuberculosis, HIV Human immune virus, CI Confidence interval, * Statistically significant at 5% significance level, OR Odds ratio, % Percentage, n Frequency, N Sample*

DISCUSSION

The current study provided valuable insights into childhood TB outcomes and factors associated with unsuccessful treatment outcomes in selected public Hospitals in Lusaka, Zambia from 2015 to 2019. The findings revealed that TB was most common in children under the age of five, who were males and were HIV-negative. Pulmonary TB was the most common type of TB. Children aged 5 to 14 years, pulmonary TB, and relapse patients had a significantly higher proportion of

bacteriologically confirmed tuberculosis. A high proportion of children who were treated for TB did not have their treatment outcomes evaluated. After the not-evaluated children were dropped, the successful TB treatment outcome fell above the WHO recommended threshold of 90, as the proportion of unsuccessful treatment outcome was relatively low. Extrapulmonary TB was significantly associated with unsuccessful treatment outcomes in children.

Pulmonary TB (75%) was found to be the most common type of TB in children, which is consistent with earlier studies (16,17). Extrapulmonary TB accounted for roughly a quarter of all children who had TB, which is consistent with findings from Zimbabwe (18), Ghana (19), and Pakistan (20). Other studies, however, found a higher proportion (33 % to 56 %) of extrapulmonary TB (16,21–23).

The proportion of bacteriologically confirmed TB among children who underwent bacteriological testing from 2015 to 2019 was 55%. This is substantially lower compared to findings from a Brazilian study (69%) (17). On the other hand, the proportion of bacteriologically confirmed TB among all children who were treated for TB from 2015 to 2019 was relatively low (11%). These findings are consistent with previous studies elsewhere (24). However, another study done in Nigeria showed a higher proportion of bacteriologically confirmed TB among all children who children being treated for TB (28%) (25).

The low proportion of bacteriologically confirmed TB among all children who were treated for TB reported in the current study might be attributed to the fact that bacteriological testing for TB was only performed on 507 children out of the total 2,531 children who were treated for TB from 2015 to 2019. This meant that the majority (89%) of children were treated for TB based on clinical diagnosis which was a combination of children who had negative bacteriological test results and

those who did not undergo bacteriological testing but might have been subjected to other means of diagnosis such as radiographs.

~~The majority (89%) of TB in children was based on clinical diagnosis. The proportion of bacteriologically confirmed TB disease was relatively low (11%), which is consistent with some previous studies elsewhere (24). However, other studies that were done in Nigeria (28%) (25) and Brazil (69%) (17) showed a higher proportion of bacteriologically confirmed cases. The low proportion of bacteriological TB reported in the current study might be attributed to the fact that bacteriological diagnostic tests were rarely performed in children treated for extrapulmonary TB, with radiographs being the most commonly used diagnostic test. Even though the introduction of Xpert MTB/RIF has improved the sensitivity of direct detection when compared to smear microscopy, bacteriological diagnosis of TB remains a challenge in children as seen from the results of the current study and the burden of TB in children will continue to be underreported until better laboratory diagnostics for children are available (26,27).~~

During the five years (2015-2019), the death outcome in children with tuberculosis was low, ranging from 1% to 3%, which was consistent with other studies (16,28). One of the primary problems in estimating the proportion of children who died from TB was that the exact causes of death were not recorded in the TB registers. As a result, the observed rates may be exaggerated if some deaths were caused by factors unrelated to TB treatment.

Treatment failure among children with TB was uncommon over the five years, and this finding was within the range (0% - 1%) which was similar to what has been reported in other studies (24).

Furthermore, the proportion of children who were lost to follow-up on TB treatment remained very low (0.4 % - 2.1 %). This was lower than what was reported in other studies in Ethiopia (16,24).

A high proportion of children with a not-evaluated treatment outcome (40.9%) was observed over the five years. In contrast to the results of this study, other studies reported a much lower rate of not-evaluated treatment outcomes (0.1% - 22.5%) (29–31).

One of the potential reasons for having a high proportion of cases with not-evaluated treatment outcomes could have been transfers that happen between higher and lower-level health facilities. In the continuum of care, patients are diagnosed from higher-level health facilities that have the necessary equipment and human resource but later get transferred to lower-level health facilities that are near the patients' homes for continuation of treatment. This has been demonstrated in literature in a study that was done in Ethiopia, which found that most children with not-evaluated treatment outcomes were transferred ~~The reported high proportion of not-evaluated treatment outcome in Hospitals may be due to patients being transferred to other health facilities, especially health facilities that are near their homes for treatment continuation after TB diagnosis, as demonstrated in one study in Ethiopia, which found that most children with not-evaluated treatment outcomes were transferred~~ (31). This was also evident in the current study as the results reviewed that over 70% of children from each of the two tertiary Hospitals (LMUTH and UTH) (Highest level facilities in Zambia) had not-evaluated treatment outcomes as compared to the six-level 1st Hospitals where the proportion of children with not-evaluated treatment outcomes was 6% to 20% with one facility reporting 38%. ~~This was also evident in the current study as the results reviewed that over 70% of children from each of the two tertiary Hospitals (LMUTH and UTH) were not evaluated for their treatment outcomes .~~

This referral system between higher and lower-level health facilities often results in a break in follow-up, making it difficult to track the progress and the success of treatment outcomes in children. This also raises questions about the guarantee of continuity of care for these children.

Additionally, apart from transfers, poor documentation, and poor follow-up systems could have also contributed to treatment outcomes not being evaluated. Without proper evaluation, it becomes difficult to determine the effectiveness and success rates of the treatments being provided.

~~This referral system between tertiary Hospitals and other health facilities often results in a break in follow-up, making it difficult to track the progress and success of treatment outcomes in children. This not only hinders the ability to track the progress of treatment but also raises questions about the guarantee of continuity of care for these children. Additionally, poor documentation and follow-up systems could also contribute to treatment outcomes not being evaluated. Without proper evaluation, it becomes difficult to determine the effectiveness and success rates of the treatments being provided.~~

After dropping the children whose treatment outcomes were not evaluated, the unsuccessful treatment outcome ranged between 2.2% to 8.1% (2015 to 2019) with a pooled estimate of 5.1%, and the successful treatment outcome ranged between 91.9% to 97.8% (2015 to 2019) with a pooled estimate of 94.9%. This showed that the successful treatment rate met the WHO-mandated 90% treatment success threshold. The not-evaluated children were dropped to avoid overestimating or underestimating successful and unsuccessful treatment outcomes. Our results were comparable to studies that reported similar successful treatment outcomes of 94.2% (Pakistan) (32), and 93.1% (Botswana) (33). However, other studies have reported lower

successful treatment outcomes 88.6% (Ethiopia) (34), 85.4% (Pakistan) (35), 83 % (Nigeria) (36), and 58.4% (Nigeria) (37).

Regression analysis revealed that extrapulmonary TB had a higher risk of unsuccessful treatment outcomes. Other studies have come to the same conclusion (21,38). The study found no association between age, sex, HIV status, treatment history, diagnostic type, and TB unsuccessful treatment outcome. Similar results have been reported in the past by some studies (20). But other studies reported that age (10,11), sex (39), HIV status (10,40,41), treatment history, and diagnostic method (39) were associated with unsuccessful treatment outcomes in children with TB. The inconclusiveness of our findings could again be because the sample size was reduced at regression analysis as the not-evaluated children could not be exclusively classified as successful or unsuccessful outcomes to reduce bias in findings of factors associated with unsuccessful treatment outcomes, hence were excluded from the regression analysis.

The strengths that were in this study include the fact that the sample included all children treated for TB between 2015 and 2019 who met the study's selection criteria, which gave the study a big sample size. However, some limitations were also noted. Documentation in the TB registers was inadequate, there was missing data (challenges of keeping records in health facilities). This might have resulted in under or overestimation of certain TB treatment outcomes. Furthermore, there was a limitation in the number of clinical and social-demographic factors that could be investigated at regression analysis as not so many variables are captured in TB registers. The large number of not-evaluated children that was observed could have also led to overestimation or underestimation of unsuccessful or successful treatment outcomes since these cases were dropped as they could not be classified as successful or unsuccessful treatment outcomes.

CONCLUSION

Pulmonary TB was the most common type of TB in children. The majority of children who were treated for TB from 2015 to 2019 were based on clinical diagnosis. Bacteriologically confirmed TB among children who were tested for TB was low. ~~Bacteriological confirmation of TB in Children was low, indicating that clinical diagnosis remained the common method of diagnosing TB in children.~~ After removing children with not-evaluated TB treatment outcomes, the treatment success rate was above the WHO's goal of 90% for the period 2015 -2019.

Children under the age of five should be targeted as a high-risk group in strategies to eliminate childhood TB/improve TB treatment success rates with a primary focus on children with extrapulmonary TB. Future studies should explore the possibility of tracking the transferred (not-evaluated) children for better estimations of TB treatment outcomes as tertiary Hospitals had a very high number of children whose outcomes were not evaluated.

Acknowledgments

The authors are grateful to The University of Zambia, School of Public Health, Department of Epidemiology and Biostatistics. We are also grateful to the Ministry of Health, Health facility management from all 8 facilities where data was collected and all health workers operating from the TB sections in the 8 health facilities that were included in this study.

References

1. World Health Organisation. Global Tuberculosis report 2022 [Internet]. Geneva; 2022. Available from: http://apps.who.int/bookorders.
2. Tuberculosis [Internet]. [cited 2023 Jan 12]. Available from: https://www.who.int/news-room/fact-sheets/detail/tuberculosis

3. World Health Organisation. GLOBAL TUBERCULOSIS REPORT 2021 [Internet]. Geneva: 2021. 2021. Available from: http://apps.who.int/bookorders.
4. Palmer AL. Advances in Pediatric Tuberculosis [Internet]. Vol. 13, Journal of Pediatric Infectious Diseases. Georg Thieme Verlag; 2018 [cited 2020 May 12]. p. 89–90. Available from: http://www.thieme-connect.de/DOI/DOI?10.1055/s-0038-1639600
5. Thomas TA. Tuberculosis in Children. Vol. 64, Pediatric Clinics of North America. W.B. Saunders; 2017. p. 893–909.
6. World Health Organization. Framework for implementing the "End TB Strategy" in the African Region 2016-2020. [Internet]. 2017. Available from: http://apps.who.int/bookorders.
7. World Health Organization. The end TB strategy. 2015;
8. World Health Organization. Roadmap towards ending TB in children and adolescents. WHO. 2018;
9. Matucci T, Galli L, de Martino M, Chiappini E. Treating children with tuberculosis: new weapons for an old enemy. https://doi.org/101080/1120009X20191598039 [Internet]. 2019 [cited 2023 Jun 30];31(5):227–45. Available from: https://www.tandfonline.com/doi/abs/10.1080/1120009X.2019.1598039
10. Mirutse G, Fang M, Kahsay AB, Ma X. Epidemiology of childhood tuberculosis and factors associated with unsuccessful treatment outcomes in Tigray, Ethiopia: A ten-year retrospective cross sectional study. BMC Public Health. 2019 Oct 24;19(1).
11. Tilahun G, Gebre-Selassie S. Treatment outcomes of childhood tuberculosis in Addis Ababa: A five-year retrospective analysis. BMC Public Health [Internet]. 2016 Jul 21 [cited 2020 May 12];16(1):612. Available from: http://bmcpublichealth.biomedcentral.com/articles/10.1186/s12889-016-3193-8
12. Jenkins HE, Yuen CM, Rodriguez CA, Nathavitharana RR, McLaughlin MM, Donald P, et al. Mortality in children diagnosed with tuberculosis: a systematic review and meta-analysis. Lancet Infect Dis. 2017 Mar 1;17(3):285–95.
13. Joshi S. A study of treatment outcome of paediatric tuberculosis patients in an urban city of central India. Int J Community Med Public Health. 2018 Mar 23;5(4):1503.
14. World Health Organization. Definitions and reporting framework for tuberculosis - 2013 revision. 2013.
15. Linh NN, Viney K, Gegia M, Falzon D, Glaziou P, Floyd K, et al. World Health Organization treatment outcome definitions for tuberculosis: 2021 update. European Respiratory Journal [Internet]. 2021 Aug 1 [cited 2023 Jul 1];58(2). Available from: https://erj.ersjournals.com/content/58/2/2100804
16. Ramos JM, Pérez-Butragueño M, Tesfamariam A, Reyes F, Tiziano G, Endirays J, et al. Comparing tuberculosis in children aged under 5 versus 5 to 14 years old in a rural hospital in southern Ethiopia: an 18-year retrospective cross-sectional study. BMC Public Health 2019 19:1 [Internet]. 2019 Jul 2 [cited 2021 Jul 7];19(1):1–9. Available from: https://bmcpublichealth.biomedcentral.com/articles/10.1186/s12889-019-7206-2
17. Carvalho ACC, da Silva Martins P, Cardoso CAA, Miceli AL, Martire T, Sant'Anna M de FBP, et al. Pediatric tuberculosis in the metropolitan area of Rio de Janeiro. International Journal of Infectious Diseases. 2020 Sep 1;98:299–304.
18. Nzombe P, Satyanarayana S, Tweya H, Timire C, Charambira K, Ncube RT, et al. Declining Trends in Childhood TB Notifications and Profile of Notified Patients in the City of Harare, Zimbabwe, from 2009 to 2018. J Trop Med. 2020;2020.

19. Ohene SA, Fordah S, Boni P. Childhood tuberculosis and treatment outcomes in Accra: A retrospective analysis. BMC Infect Dis. 2019 Aug 28;19(1).
20. Brooks MB, Malik A, Khan S, Ahmed JF, Siddiqui S, Jaswal M, et al. Predictors of unsuccessful tuberculosis treatment outcomes in children from a prospective cohort study in Pakistan. J Glob Health [Internet]. 2021 [cited 2021 Aug 3];11:1–8. Available from: /pmc/articles/PMC7916443/
21. Abdullah A, Ahmad N, Atif M, Khan S, Wahid A, Ahmad I, et al. Treatment Outcomes of Childhood Tuberculosis in Three Districts of Balochistan, Pakistan: Findings from a Retrospective Cohort Study. J Trop Pediatr [Internet]. 2020 Jul 10 [cited 2021 Jul 28]; Available from: https://academic.oup.com/tropej/advance-article/doi/10.1093/tropej/fmaa042/5869556
22. Ogbudebe C, Adepoju V, Ekerete-Udofia C, Abu E, Egesemba G, Chukwueme N. Childhood Tuberculosis in Nigeria: Disease Presentation and Treatment Outcomes. Health Serv Insights. 2018;11.
23. Khantee P, Laoprasopwattana K. Comparing Treatment Outcomes between Confirmed and Non-Confirmed Childhood TB Cases: Study from a Major Tertiary Center in Thailand. J Trop Pediatr [Internet]. 2021 [cited 2021 Aug 8];67(1). Available from: https://academic.oup.com/tropej/article/67/1/fmaa125/6075108
24. Abdullah A, Ahmad N, Atif M, Khan S, Wahid A, Ahmad I, et al. Treatment Outcomes of Childhood Tuberculosis in Three Districts of Balochistan, Pakistan: Findings from a Retrospective Cohort Study. J Trop Pediatr [Internet]. 2021 Jul 2 [cited 2021 Aug 7];67(3). Available from: https://academic.oup.com/tropej/article/67/3/fmaa042/5869556
25. Ogbudebe CL, Adepoju V, Ekerete-Udofia C, Abu E, Egesemba G, Chukwueme N, et al. Childhood Tuberculosis in Nigeria: Disease Presentation and Treatment Outcomes. Health Serv Insights [Internet]. 2018 [cited 2023 Jun 30];11. Available from: https://pubmed.ncbi.nlm.nih.gov/29511357/
26. Dunn JJ, Starke JR, Revell PA. Laboratory diagnosis of mycobacterium tuberculosis infection and disease in children. J Clin Microbiol. 2016 Jun 1;54(6):1434–41.
27. Gutiérrez-González LH, Juárez E, Carranza C, Carreto-Binaghi LE, Alejandre A, Cabello-Gutiérrrez C, et al. Immunological Aspects of Diagnosis and Management of Childhood Tuberculosis. Infect Drug Resist [Internet]. 2021 [cited 2021 Nov 22];14:929. Available from: /pmc/articles/PMC7955028/
28. Laghari M, Sulaiman SAS, Khan AH, Memon N. A prospective study of socio-demographic, clinical characteristics and treatment outcomes of children with tuberculosis in Sindh, Pakistan. BMC Infect Dis [Internet]. 2019 Jan 24 [cited 2020 Aug 22];19(1):1–11. Available from: https://link.springer.com/articles/10.1186/s12879-019-3702-3
29. Abdullah A, Ahmad N, Atif M, Khan S, Wahid A, Ahmad I, et al. Treatment Outcomes of Childhood Tuberculosis in Three Districts of Balochistan, Pakistan: Findings from a Retrospective Cohort Study. J Trop Pediatr [Internet]. 2021 Jul 2 [cited 2021 Aug 7];67(3). Available from: https://academic.oup.com/tropej/article/67/3/fmaa042/5869556
30. Hamid M, Brooks MB, Madhani F, Ali H, Naseer MJ, Becerra M, et al. Risk factors for unsuccessful tuberculosis treatment outcomes in children. EHTESHAM HS, editor. PLoS One [Internet]. 2019 Sep 25 [cited 2020 May 12];14(9):e0222776. Available from: http://dx.plos.org/10.1371/journal.pone.0222776

31. Mirutse G, Fang M, Kahsay AB, Ma X. Epidemiology of childhood tuberculosis and factors associated with unsuccessful treatment outcomes in Tigray, Ethiopia: A ten-year retrospective cross sectional study. BMC Public Health. 2019 Oct 24;19(1).
32. Brooks MB, Malik A, Khan S, Ahmed JF, Siddiqui S, Jaswal M, et al. Predictors of unsuccessful tuberculosis treatment outcomes in children from a prospective cohort study in Pakistan. J Glob Health [Internet]. 2021 [cited 2023 Jul 2];11:1–8. Available from: /pmc/articles/PMC7916443/
33. Siamisang K, Rankgoane-Pono G, Madisa TM, Mudiayi TK, Tlhakanelo JT, Mubiri P, et al. Pediatric tuberculosis outcomes and factors associated with unfavorable treatment outcomes in Botswana, 2008–2019: a retrospective analysis. BMC Public Health [Internet]. 2022 Dec 1 [cited 2023 Jul 2];22(1):1–8. Available from: https://bmcpublichealth.biomedcentral.com/articles/10.1186/s12889-022-14477-y
34. Weldegebreal F, Teklemariam Z, Mitiku H, Tesfaye T, Abrham Roba A, Tebeje F, et al. Treatment outcome of pediatric tuberculosis in eastern Ethiopia. Front Pediatr. 2022 Aug 11;10:966237.
35. Hamid M, Brooks MB, Madhani F, Ali H, Naseer MJ, Becerra M, et al. Risk factors for unsuccessful tuberculosis treatment outcomes in children. PLoS One [Internet]. 2019 Sep 1 [cited 2023 Jul 2];14(9). Available from: /pmc/articles/PMC6760830/
36. Ogbudebe CL, Adepoju V, Ekerete-Udofia C, Abu E, Egesemba G, Chukwueme N, et al. Childhood Tuberculosis in Nigeria: Disease Presentation and Treatment Outcomes. https://doi.org/101177/1178632918757490 [Internet]. 2018 Feb 22 [cited 2023 Jul 2];11. Available from: https://journals.sagepub.com/doi/10.1177/1178632918757490
37. Alex-Hart BA, Paul NI, Alex-Hart BA, Paul NI. Pattern and Outcome of Childhood Tuberculosis Seen at the University of Port Harcourt Teaching Hospital, Nigeria. J Tuberc Res [Internet]. 2019 Sep 3 [cited 2023 Jul 2];7(3):170–83. Available from: http://www.scirp.org/journal/PaperInformation.aspx?PaperID=95501
38. Li T, Ma Y, Liu K, Shewade HD, Zhang H, Du X, et al. Childhood TB in China: notification, characteristics and risk factors for outcomes, 2010–2017. International Journal of Tuberculosis and Lung Disease. 2020 Dec 1;24(12):1285–93.
39. Hamid M, Brooks MB, Madhani F, Ali H, Naseer MJ, Becerra M, et al. Risk factors for unsuccessful tuberculosis treatment outcomes in children. EHTESHAM HS, editor. PLoS One [Internet]. 2019 Sep 25 [cited 2020 May 12];14(9):e0222776. Available from: http://dx.plos.org/10.1371/journal.pone.0222776
40. Osman M, Lee K, du Preez K, Dunbar R, Hesseling AC, Seddon JA, et al. EXCELLENT TREATMENT OUTCOMES IN CHILDREN TREATED FOR TUBERCULOSIS UNDER ROUTINE OPERATIONAL CONDITIONS IN CAPE TOWN AUTHORS. 2017;
41. Belay GM, Wubneh CA. Childhood tuberculosis treatment outcome and its association with HIV co-infection in Ethiopia: A systematic review and meta-analysis [Internet]. Vol. 48, Tropical Medicine and Health. BioMed Central Ltd.; 2020 [cited 2020 Aug 28]. Available from: /pmc/articles/PMC7027074/?report=abstract

PGPH-D-23-01445

Childhood Tuberculosis Outcomes and Factors Associated with Unsuccessful Treatment Outcomes in Selected Public Hospitals of Lusaka Zambia from 2015 to 2019. PLOS Global Public Health

Response To Reviewers' Comments

Key

- Reviewers' comments – blue color
- Authors' responses – black color
- Line numbers, tables, and figures for reference – red color
- The line numbers in this document correspond to the revised manuscript with no track changes.

Reviewer #2:

1. Only 11% of the children yielded positive bacteriological results. Establishing and reporting the denominator is crucial, specifically for children who underwent bacteriologic testing. Including children who did not undergo microbiological tests in the denominator would be misleading.

Thank you very much. Revisions have been made. Table 2 has been redone and we now have the denominator for the children who underwent microbiological testing for TB. Out of the total 2,531 children, bacteriological tests for TB were performed on 20.0% (507/2531) of children, of whom, 55% (279/507) had a positive test result and 45% (288/507) had negative test results. The 2,252 children who did not undergo bacteriological testing have been removed from the denominator when estimating the proportion of children who yielded positive bacteriological results (Table 2) Lines (260 – 261)

The 11% (279/2531) now represents the proportions of children who received TB treatment after a bacteriological confirmation of TB among all children who were treated for TB from 2015 to 2019 in the 8 health facilities. This shows that the remaining 89% (2,252/2,531) received TB treatment based on clinical diagnosis. Children who were treated for TB clinically (2,252) comprised of

children who had negative bacteriological results (228) and those who did not undergo bacteriological testing (2,024). (Table 2) (Figure 1) Lines (270 - 273)

We have also revised our discussion Lines (346 – 359)

2. A significant proportion of children with extra-pulmonary TB (EPTB) did not undergo bacteriological testing, necessitating clarification of the denominator. Among children with EPTB who underwent bacteriological testing, please confirm those with confirmed bacteriological TB.

Revisions made. The above statement has been rewritten. This is because Table 2 where this information came from has been redone. Table 2 now shows the overall denominator for children who underwent bacteriological testing which was 507.

The new Table 2 now reviews that out of 507 children who underwent bacteriological testing, 441 had pulmonary TB while only 64 had extra-pulmonary TB. There was a statistically significantly higher proportion of children with pulmonary TB 60.1% (265/441) who had a positive bacteriological test result compared to children with extrapulmonary TB 21.9% (14/64). (Table 2) Line (264 – 266; 279 - 281)

3. In the discussion section, the authors omitted to mention other potential reasons for not evaluating treatment outcomes, as recommended by the second reviewer. For instance, if transfers occurred, it might be beneficial to say the reasons behind these transfers, considering that this constitutes a substantial group of children diagnosed with TB who are lost within the healthcare system. As previously suggested, including the baseline characteristics of this group separately would be valuable.

Revisions have been made. Transfers were suspected to be one of the main reasons for a high number of not evaluated children. This is because in the continuum of care, patients are diagnosed from higher-level health facilities that have the necessary equipment and human resources but later get transferred to lower-level health facilities that are near the patients' homes for continuation of treatment. This was evident in the current study as the results reviewed that over 70% of children from each of the two tertiary Hospitals (LMUTH and UTH) (Highest level facilities in Zambia) had not-evaluated treatment outcomes as compared to the six-level 1st Hospitals where the proportion of children with not-evaluated treatment outcomes was 6% to 20% with one facility reporting 38%. Line (369 – 382)

Additionally, apart from transfers other potential reasons for a high number of not-evaluated cases such as poor documentation, and poor follow-up systems have also been added to the discussion. Line (383 – 388)

We have also included a new table showing the baseline characteristics of children who did not have treatment outcomes evaluated against those who had their treatment outcomes evaluated (Supplementary Table 1).

4. Consider incorporating a flow diagram to illustrate the number of children investigated for TB, those excluded, the status of bacteriological tests performed, and other relevant details. This visual aid can enhance the clarity of the text.

Revisions have been made. A study flow diagram has been added. Narrations have also been added to explain the study flow chart. The study was done on children who were treated for TB based on records from the TB registers. The TB registers contained records of all the children who were treated for TB from 2015 to 2019 based on bacteriological confirmation of TB or clinical diagnosis (Figure 1) Lines (160 – 169; 185 – 221).
