# Supplementary material for: Childhood tuberculosis outcomes and factors associated with unsuccessful treatment outcomes in selected public hospitals of Lusaka Zambia from 2015 to 2019
Source: PLOS Glob Public Health. 2024 Oct 11;4(10):e0002591. doi: 10.1371/journal.pgph.0002591 (PMC11469523; doi:10.1371/journal.pgph.0002591)
Supplement: Attachment — Submitted filename: Response to Reviewers.docx [file pgph.0002591.s002.docx]

**PGPH-D-23-01445**
Childhood Tuberculosis Outcomes and Factors Associated with Unsuccessful Treatment Outcomes in Selected Public Hospitals of Lusaka Zambia from 2015 to 2019.
PLOS Global Public Health

**Response To Reviewers’ Comments**

Key

- Reviewers’ comments – blue color
- Authors' responses – black color
- Line numbers, tables, and figures for reference – red color
- The line numbers in this document correspond to the revised manuscript with hidden track changes.

**Additional Editor Comments (if provided):**

Please explain the rationale for selecting the hospitals included and where they are situated, urban, rural- could outcomes differ by this geolocation urban vs rural or by hospital? This should be investigated.

The study was conducted in all Public Level 1 Hospitals and University Teaching Hospitals. This gave a total of 8 facilities and all of them were within the urban City of Lusaka, Zambia.

Level 1 Hospitals and University Teaching Hospitals have the capacity to conduct clinical and bacteriological confirmation of TB in children, as they are equipped with laboratory and radiological tests as well as specialized medical personnel and act as referral centers for all clinics in the city of Lusaka. Revisions have been made. Line (89 - 97).

Wasn't there more information about these children? other comorbid conditions other than TB

The study was done retrospectively using records from TB registers only. Information on other comorbid conditions was not found in the TB registers because it was not documented. This has now been added as a limitation in our study. Line (329 - 331).

Line 347 can the authors explain this ....diagnosed clinically, making it a challenge to evaluate the true magnitude of TB disease in children before investigating the treatment outcomes..... , since it is known that TB in young children in is paucibacillary and will have to be diagnosed clinically, and that sputum sample collection is challenge in children

Correction has been made, the statement in line 347 has been revised Line (336 - 338)

Who are those not evaluated? Are they the same as the rest of the sample analyses. This a major finding that may children have no outcomes- is this across all area/ hospitals??

Not evaluated – A TB patient for whom no treatment outcome is assigned. This includes cases ‘transferred out’ to another treatment unit as well as cases for whom the treatment outcome is unknown to the reporting unit (Line 128 – 130).

The not evaluated children were part of the first analysis which includes the demographic characteristics as well as the TB diagnosis. But they were excluded in the analysis for successful and unsuccessful treatment outcome and the regression analysis because their actual outcome remained unknown since it was not document.

Yes, it was a major finding looking at the fact that the proportion was high. We have made revisions and included this in the abstract, results, conclusion and discussed what could have possibly led to that (Line 44; 48 – 49; 213 – 215; 290 – 305; 342 – 344)

Additionally, the distribution of not-evaluated children was not the same across the health facilities. The largest proportion of children whose treatment outcome was not evaluated was observed in the 2 tertiary institutions. A table of showing treatment outcomes across all the facilities has been added and this finding has been discussed (Table 3) (Line 218 – 220; 290 – 305; 342 – 344)

**Reviewer #1:** 
I have three major concerns with this manuscript:
1. Although the authors are reporting treatment outcomes and factors associated with these outcomes, the analysis and discussion is limited and no new information or insights are presented. I am aware that this is probably due to the limited data captured in the TB registers but wondered if any additional information could be presented. For example, was the type of extrapulmonary TB reported?

Yes, the amount of data captured in the TB registers was a limitation on the number of factors that we could investigate in this study (Line 329 - 331. The type of extrapulmonary TB was also not part of the information that we could find in the TB registers.

But we have revised our discussion. We have added more new information on how TB outcomes varied between pulmonary and extrapulmonary TB and how treatment outcomes by health facility (Table 3) (Line 216 – 220; 231 – 233; 260 – 263; 290 - 314)

2. In lines 251 – 256, table 3, and the conclusion the authors state that over 90% of the children had a successful treatment outcome. This is not exactly correct and overstating things, as it is only if the 41% of the children ‘not evaluated’ are excluded that the successful treatment outcome rate is over 90%. This needs to be clarified in the abstract, manuscript, and table.

We have made the corrections. The overstatements have all been revised in the abstract, results, discussions, and conclusion sections to clarify that the, “over 90% successful treatment outcome” was got after the ‘Not evaluated’ children were excluded (Line 44 - 46; 48 - 49; 225 - 233; 261-262; 306 – 314; 338 - 339)

3. As mentioned in point 2 above, the pooled proportion of children for whom no treatment outcome was assigned is 41%. In other words, a little less than half of the children had no treatment outcome assigned. This is a very large proportion of children not evaluated and must be mentioned in the **abstract and the results and not just in the third last paragraph of the discussion.**

In the paragraph in which this is discussed, the authors suggest that this large number could be as patients are transferred to another treatment facility. However, there are other reasons as to why no treatment outcome is assigned, mostly because of lost contact with patients. These would include treatment interruption, sub-optimal adherence and even death. Are there any other journal articles that discuss reasons for ‘not evaluated’? Given the extent of the children in the not evaluated' category, this is something the authors could include in the discussion. That is the limitation of this indicator.
Yes, it was a major finding looking at the fact that the proportion was high. We have made revisions and included this in the abstract, results, conclusion and discussed what could have possibly led to that and the implications that it has on the estimation of treatment outcomes (Line 44; 48 – 49; 213 – 215; 290 – 314; 329 – 331; 342 – 344)

Additionally, the distribution of not-evaluated children was not the same across the health facilities. The largest proportion of children whose treatment outcome was not evaluated was observed in the 2 tertiary institutions. A table showing treatment outcomes across all the facilities has been added and this finding has been discussed (Table 3) (Line 218 – 220; 290 – 305; 342 – 344).

Additional comments:
Abstract:
1. ‘A total enumeration of children aged 0-14 years who were diagnosed and treated for tuberculosis between 2015 and 2019 was done.’
The sentence above does not make sense. Do you mean ‘All children 0 – 14 years who were diagnosed and treated for TB between 2015 and 2019 were included’?
Corrections have been made (Line 32 – 34)

2. ‘Overall, (6.3%) of patients were cured, (49.7%) completed treatment, (0.9%) lost to follow-up, (1.8%) died, 0.3% failed treatment and 40.9% were not evaluated.’
In the sentence above the proportion of patients should not be in brackets.

Corrections have been made (Line 43 – 45)

‘Children with extrapulmonary tuberculosis were associated with unsuccessful treatment outcomes (AOR 1.64; 95 % CI: 1.02 – 2.62).
The sentence above should start with ‘Extrapulmonary…’.
Corrections have been made (Line 46 – 47)

Introduction
Line 77: Some of the formatting has got lost on the way. I assume this should be a new paragraph?

Revisions have been made (Line 76 – 78)

Line 78: Replace ‘would be’ with ‘is’.

Corrections have been made (Line 80)

Line 89: ‘…might help in the continuation of making informed decisions…’
Rather ‘will provide evidence to inform decisions’

Corrections have been made (Line 82 - 83)

Methods
Line 88: In this line and lines 98 and 158 you say that you looked at files and in line 100 and somewhere else you say you captured data from the TB registers. Please address this inconsistency. I can’t believe you looked at over 2000 files!

Data for the study was extracted from TB registers and not patients’ files. Corrections have now been to address this inconsistency (Line 89; 102; 108)

Line 93: You give inclusion and exclusion criteria for the children, but you also need to explain your rationale for your choice of health facilities and address whether this could have introduced some bias.

Revisions have been made to the study settings’ rationale. The study was conducted in all Public Level 1 Hospitals and University Teaching Hospitals within the urban City of Lusaka, Zambia. This comprised 6 level 1 Hospitals (Chawama, Chelstone, Chipata, Chilenje, Kanyama, and Matero) and 2 University Teaching Hospitals (Levi Mwanawasa University Teaching Hospital (LMUTH) and University Teaching Hospital (UTH)). Therefore the number 8 was comparised of all the level 1 and university teaching hospitals in urban Lusaka.

These facilities are hospitals and operate as referral facilities for clinics and they have the capacity to conduct clinical and bacteriological confirmation of TB in children, as they are equipped with laboratory and radiological tests as well as specialized medical personnel (Line 89 – 97)

Line 100: ‘The study employed a total renumeration of all the data..’ This sentence is very confusing and it is unclear exactly what you are trying to say.

Corrections have been made (Line 104 – 106)

Line 161: We try not to use the terminology ‘TB cases’ anymore but rather something like ‘people with TB’ or ‘patients with TB.’

Corrections have been made. “TB cases” has been changed to “Children that had TB” (Line 166)

Results
Much of the text in the results section repeats what is in the table. Usually, just a couple of significant results from the table are highlighted in the text.
Line 264: Do you need to put a p-value if there is also a confidence interval?

Revisions and corrections have been made to the narrations in the results section (Line 205 – 235). The p-value in line 264 has been corrected to now (Line 245 – 246)

Discussion
Line 325: Replace ‘investigations’ with ‘studies’.

Corrections have been made (Line 316)

**Reviewer #2:**

This is a very interesting and well-written manuscript addressing a critical problem of Childhood Tuberculosis Outcomes and Factors Associated with Unsuccessful Treatment Outcomes. I have a few comments:
1. in the abstract: L35 "treatment completed", remove the comma in between

Corrections have been made (Line 35)

2.L56 The number of new TB cases were "estimated" and not "anticipated"

Corrections have been made (Line 57)

3. Can the authors clarify the criteria for selecting the 8 Healthcare facilities

Revisions have been made to the study setting. The study was conducted in all Public Level 1 Hospitals and University Teaching Hospitals within the urban City of Lusaka, Zambia. This comprised 6 level 1 Hospitals (Chawama, Chelstone, Chipata, Chilenje, Kanyama, and Matero) and 2 University Teaching Hospitals (Levi Mwanawasa University Teaching Hospital (LMUTH) and University Teaching Hospital (UTH)). Therefore, the number 8 was comparised of all the level 1 and university teaching hospitals in urban Lusaka.

These facilities are hospitals and operate as referral facilities for clinics and they have the capacity to conduct clinical and bacteriological confirmation of TB in children, as they are equipped with laboratory and radiological tests as well as specialized medical personnel (Line 89 – 97)

4.L189: add the number in ()in addition to % for all results so we can see the numerator/denominator

Corrections made for all the narrations throughout the results section (Line 160 – 235)

5.L193: add% at 11.8 for female

Corrections made (Line 189)

6. it might be useful to show the disaggregated treatment outcomes per Type of TB( pulmonary /extrapulmonary), as the main result of the study has shown that extra pulmonary TB is associated with unsuccessful treatment outcome. How many children (%) with extrapulmonary TB died? were Lost to follow-up or failed treatment?

Revisions have been made. An additional table has been added showing treatment outcomes by type of TB (Table 3) (Line 231 – 233; 236 - 238)

7. As 41% of children did not have a TB treatment outcome, how many had pulmonary and extrapulmonary TB? It might be useful to provide the characteristics of this particular group. Are they different compared to those with a treatment outcome?

Revisions have been made, the 41% standing for not evaluated children has also been disaggregated by type of TB (Table 3).

The table reviewed that 38% of those who had extra pulmonary TB were not evaluated as compared to 48% of those with pulmonary TB were not evaluated. However, what we noted was that of the 41% of not evaluated children, most of them came from tertiary hospitals. Above 70% of children in each of the 2 tertiary hospitals (UTH and LMUTH) were not evaluated. We have added this new information and discussed it (Line 216 – 220)
