# Supplementary material for: Childhood tuberculosis outcomes and factors associated with unsuccessful treatment outcomes in selected public hospitals of Lusaka Zambia from 2015 to 2019
Source: PLOS Glob Public Health. 2024 Oct 11;4(10):e0002591. doi: 10.1371/journal.pgph.0002591 (PMC11469523; doi:10.1371/journal.pgph.0002591)
Supplement: Attachment — Submitted filename: Response to Reviewers.docx [file pgph.0002591.s003.docx]

**PGPH-D-23-01445**
Childhood Tuberculosis Outcomes and Factors Associated with Unsuccessful Treatment Outcomes in Selected Public Hospitals of Lusaka Zambia from 2015 to 2019.
PLOS Global Public Health

**Response To Reviewers’ Comments**

***Key***

- Reviewers’ comments – blue color
- Authors' responses – black color
- Line numbers, tables, and figures for reference – red color
- The line numbers in this document correspond to the revised manuscript with no track changes.

**Reviewer #2:**

1. Only 11% of the children yielded positive bacteriological results. Establishing and reporting the denominator is crucial, specifically for children who underwent bacteriologic testing. Including children who did not undergo microbiological tests in the denominator would be misleading.

Thank you very much. Revisions have been made. Table 2 has been redone and we now have the denominator for the children who underwent microbiological testing for TB. Out of the total 2,531 children, bacteriological tests for TB were performed on 20.0% (507/2531) of children, of whom, 55% (279/507) had a positive test result and 45% (288/507) had negative test results. The 2,252 children who did not undergo bacteriological testing have been removed from the denominator when estimating the proportion of children who yielded positive bacteriological results (Table 2) Lines (260 – 261)

The 11% (279/2531) now represents the proportions of children who received TB treatment after a bacteriological confirmation of TB among all children who were treated for TB from 2015 to 2019 in the 8 health facilities. This shows that the remaining 89% (2,252/2,531) received TB treatment based on clinical diagnosis. Children who were treated for TB clinically (2,252) comprised of children who had negative bacteriological results (228) and those who did not undergo bacteriological testing (2,024). (Table 2) (Figure 1) Lines (270 - 273)

We have also revised our discussion Lines (346 – 359)

2. A significant proportion of children with extra-pulmonary TB (EPTB) did not undergo bacteriological testing, necessitating clarification of the denominator. Among children with EPTB who underwent bacteriological testing, please confirm those with confirmed bacteriological TB.

Revisions made. The above statement has been rewritten. This is because Table 2 where this information came from has been redone. Table 2 now shows the overall denominator for children who underwent bacteriological testing which was 507.

The new Table 2 now reviews that out of 507 children who underwent bacteriological testing, 441 had pulmonary TB while only 64 had extra-pulmonary TB. There was a statistically significantly higher proportion of children with pulmonary TB 60.1% (265/441) who had a positive bacteriological test result compared to children with extrapulmonary TB 21.9% (14/64). (Table 2) Line (264 – 266; 279 - 281)

3. In the discussion section, the authors omitted to mention other potential reasons for not evaluating treatment outcomes, as recommended by the second reviewer. For instance, if transfers occurred, it might be beneficial to say the reasons behind these transfers, considering that this constitutes a substantial group of children diagnosed with TB who are lost within the healthcare system. As previously suggested, including the baseline characteristics of this group separately would be valuable.

Revisions have been made. Transfers were suspected to be one of the main reasons for a high number of not evaluated children. This is because in the continuum of care, patients are diagnosed from higher-level health facilities that have the necessary equipment and human resources but later get transferred to lower-level health facilities that are near the patients' homes for continuation of treatment. This was evident in the current study as the results reviewed that over 70% of children from each of the two tertiary Hospitals (LMUTH and UTH) (Highest level facilities in Zambia) had not-evaluated treatment outcomes as compared to the six-level 1st Hospitals where the proportion of children with not-evaluated treatment outcomes was 6% to 20% with one facility reporting 38%. Line (369 – 382)

Additionally, apart from transfers other potential reasons for a high number of not-evaluated cases such as poor documentation, and poor follow-up systems have also been added to the discussion. Line (383 – 388)

We have also included a new table showing the baseline characteristics of children who did not have treatment outcomes evaluated against those who had their treatment outcomes evaluated (Supplementary Table 1).

4. Consider incorporating a flow diagram to illustrate the number of children investigated for TB, those excluded, the status of bacteriological tests performed, and other relevant details. This visual aid can enhance the clarity of the text.

Revisions have been made. A study flow diagram has been added. Narrations have also been added to explain the study flow chart. The study was done on children who were treated for TB based on records from the TB registers. The TB registers contained records of all the children who were treated for TB from 2015 to 2019 based on bacteriological confirmation of TB or clinical diagnosis (Figure 1) Lines (160 – 169; 185 – 221).
