# Supplementary material for: Childhood tuberculosis outcomes and factors associated with unsuccessful treatment outcomes in selected public hospitals of Lusaka Zambia from 2015 to 2019
Source: PLOS Glob Public Health. 2024 Oct 11;4(10):e0002591. doi: 10.1371/journal.pgph.0002591 (PMC11469523; doi:10.1371/journal.pgph.0002591)
Supplement: Attachment — Submitted filename: Response to Reviewers.docx [file pgph.0002591.s005.docx]

**PGPH-D-23-01445**
Childhood Tuberculosis Outcomes and Factors Associated with Unsuccessful Treatment Outcomes in Selected Public Hospitals of Lusaka Zambia from 2015 to 2019.
PLOS Global Public Health

**Response To Reviewers’ Comments**

***Key***

- Reviewers’ comments – blue color
- Authors' responses – black color
- Line numbers, tables, and figures for reference – red color
- The line numbers in this document correspond to the revised manuscript with no track changes.

**Reviewer #3:**

Why delay the publication?

The work presented in this manuscript was part of the authors’ academic work outputs for a research project between (2020 to 2023). The data collection process (data extraction from TB registers) was conducted in the first quarter of 2021. Data entry, cleaning, and analysis were done in the same year 2021 but the writing of the primary manuscript was performed in 2022. We then submitted the manuscript for peer review and possible publication to PLOS Global Public Health in July 2023.

Cohort?

This study was a cross-sectional study that involved the extraction of secondary data from TB registers from the eight hospitals. Revisions have been made (Line 31).

Eight?

Corrections have been made (Line 31)

EPTB will usually have treatment completed…how were other outcomes measured?

The results showed that not everyone with EPTB had treatment completed. Out of 631 children who had EPTB, only 53.7% (339) had treatment completed (Table 3)

Revisions have been made; all treatment outcomes were measured based on the standard WHO definitions of TB treatment outcomes. We have provided details under the methodology (Lines 115 - 131)

How many? What percentage?

Out of 2531 children who were treated for TB between 2015 and 2019, 49% did not have their treatment outcomes evaluated. Revisions have been made (Line 47)

Not evaluated reasons can be cited in the results

Thank you very much for the comment, we were just not sure of the exact meaning of the comment. However, this is what we have done, the reasons for having a higher number of not-evaluated children have been put under the discussion (Lines 336 - 352)

Furthermore, the statement “The successful treatment outcome met the World Health Organization's threshold goal of 90% after children who were not 49 evaluated were dropped” implied that the successful treatment rate should not be generalized to all the children who were treated for TB between 2015 and 2019, but should only apply to those who had their treatment outcomes evaluated (Lines 47 – 49)

Repeated; can use children instead or early adolescence

Corrections have been made, we have removed treatment outcomes and used children as one of the keywords (Line 51)

Cohort?

This study was a cross-sectional study that involved the extraction of secondary data from TB registers from all eight hospitals. Corrections have been made (Line 87)

Eight

Corrections have been made (Line 98)

Can mention the percentage also

Revisions have been made; percentages have been added (Lines 167 – 169)

Can mention the most common age group, if locally relevant only then the hospital's proportions maybe mentioned.

Revisions have been made (Lines 185 – 186). Proportions for the most common age groups have been mentioned. Additionally, it is of local relevance that we present the proportions for the hospitals, this is because UTH and LMUTH are the biggest hospitals in Zambia as they serve as tertiary hospitals, while the other 6 hospitals in this study were level 1 hospitals.

Because of the vast difference in the absolute numbers in the two groups, there may be an overestimation in the statistical significance. This may be brought out in the discussion.

Revisions have been made, (Line 382 – 385)

Not evaluated may be removed from the denominator, and the cured, died, etc. outcomes may be expressed.

Revisions have been made. Not evaluated children were removed from the denominator and all outcomes were recalculated (Table 3) and narrations edited (Lines 41- 46; 256 – 227).

Trend analysis for the category of outcomes may be shown in figures

Revisions have been made. Trends have been presented using graphs (Fig 2 and Fig 3).

What were the confounders based on the adjusted analysis? Can be discussed

The study had few variables that could be controlled for as confounders under regression analysis. This is because the study was based on data extracted from TB registers where not so many variables are documented. This limitation has been put in the discussion (Line 377 – 379).

Under adjusted analysis, the investigator-led-stepwise logistic regression model came out as the best-fit model, of which only 5 variables made it to this best-fit model. These were Age group, Sex, Types of TB HIV status, and Diagnostic type. These variables have been discussed (Line 363 – 372)
