# Supplementary material for: Childhood tuberculosis outcomes and factors associated with unsuccessful treatment outcomes in selected public hospitals of Lusaka Zambia from 2015 to 2019
Source: PLOS Glob Public Health. 2024 Oct 11;4(10):e0002591. doi: 10.1371/journal.pgph.0002591 (PMC11469523; doi:10.1371/journal.pgph.0002591)
Supplement: Attachment — Submitted filename: Response to Reviewers.docx [file pgph.0002591.s006.docx]

**PGPH-D-23-01445**
Childhood Tuberculosis Outcomes and Factors Associated with Unsuccessful Treatment Outcomes in Selected Public Hospitals of Lusaka Zambia from 2015 to 2019.
PLOS Global Public Health

**Response To Reviewers’ Comments**

***Key***

- Reviewers’ comments – blue color
- Authors' responses – black color
- Line numbers, tables, and figures for reference – red color
- The line numbers in this document correspond to the revised manuscript with no track changes.

Additional Editor Comments (if provided):

1. Introduction can include

a. the incidence of TB in Zambia, and

b. what proportion of all TB cases in Zambia are in the pediatric age group?

Revisions have been made **(Lines 57 - 60).**

2. Setting description should include

a. The various level hospitals in Zambia with the population covered and what is meant by level 1.

Revisions have been made. In Zambia, First-level hospitals are the third-largest levels of care after the Second and Third-level hospitals. They provide diagnostic, and clinical services in support of health center referrals. On the other hand, Third-level hospitals otherwise known as University Teaching Hospitals or Tertiary hospitals are the highest referral hospitals in Zambia. All complicated cases not attended to at First or Second-level hospitals are referred to Third-level Hospitals.

Both first-level and Third-level hospitals, conduct clinical and bacteriological diagnoses of TB in children, as they are equipped with laboratory and radiological tests as well as specialized medical personnel and act as referral Hospitals for all health centers in the city of Lusaka **(Lines 96 - 104).**

b. Details of the treatment given should be mentioned as there was a shift from alternate-day regimen to daily regimen in many countries during the time of this study.

Thank you very much for the comment. In the files where the data was extracted, there was missing information concerning details of the type of drugs/regime that the children were taking. The only information that was recorded in a good manner was Treatment Type, where we had; Initial regimen with first-line drug, Retreatment regimen with first-line drug, and Second-line treatment drug **(Table 1).** We had noted that this was a limitation in our study and we have included it under the limitation section of our study **(Table 1) (Line 184 – 185; 364 - 365).**

3. How many were the total cases in the registers? What proportion of all cases had missing information and therefore excluded to reach 2531?

The total number of files extracted was 2,531. Out of these 1,036 files had missing information on treatment outcome. These have been excluded from the analysis. Revisions have been made **(Fig 1).**

4. A large proportion (40.9%) of all cases (2531) included in the study did not have their outcomes evaluated. This study is about describing the treatment outcomes of paediatric TB and identifying factors associated with it. Therefore the number of cases to be included for this study should be 1495, for whom the treatment outcome is available, and not 2531. The demographic and clinical characteristics should be given for the 1495 cases. Add a column in table 1 for the 1495 who had treatment outcomes. Please modify the text accordingly.

Thank you very much. Revisions have been made. Since we were describing the treatment outcomes of pediatric TB and identifying factors associated with it, the total number of study participants has been reduced to 1,495 for whom treatment outcomes were evaluated. Table 1 has been adjusted accordingly including the study flow chart Narrations have also been adjusted accordingly in the abstract, results, and discussion **sections (Fig 1) (Table 1) (Lines 169 – 173; 177 – 185).**

Further information on the profile for 1036 children with missing information on treatment outcomes has been provided in the supplementary table **(Table S1).**

5. Bacteriological confirmation would be relevant for interpreting the treatment outcomes and therefore this description should be provided for the 1495. Description of 2531 cases and the differences in the bacteriological positivity between the groups is not as per the objectives of the study. Replace the denominator for the table 2 with 1495. Please modify the text accordingly.

Revisions have been made. Table 2 for bacteriological confirmation of TB has been redone using 1,495 as people that had treatment outcomes. Narrations have also been adjusted accordingly in the results section as well as the discussion **(Table 2) (Line 202 – 217).**

6. Discussion should include

a. what proportion of the total paediatric TB cases in Zambia were included in this study?

b. to what extent they were representative of all the cases?

c. the differences between 2531 and 1495 in terms of their clinical characteristics.

Based on table 1. We noticed that there wasn’t much difference between 2531 (all children) and 1495 (With evaluated treatment outcomes) in terms of clinical characteristics. Revisions have been made accordingly **(Table 1) (Lines 359 – 361).**

Our study only focused at TB cases in children from 8 hospitals only within the city of Lusaka, Zambia. Therefore, the results presented here are not representative of all the TB cases in children in the city of Lusaka or Zambia at large. We have made revisions and added this limitation to the discussion **(Lines 351 - 352).**
